# Dersimelagon, a novel oral melanocortin 1 receptor agonist, demonstrates disease-modifying effects in preclinical models of systemic sclerosis

**DOI:** 10.1186/s13075-022-02899-3

**Published:** 2022-09-01

**Authors:** Masahiro Kondo, Tsuyoshi Suzuki, Yuko Kawano, Shinji Kojima, Masahiko Miyashiro, Atsuhiro Matsumoto, Gabriela Kania, Przemysław Błyszczuk, Rebecca L. Ross, Panji Mulipa, Francesco Del Galdo, Yun Zhang, Jörg H. W. Distler

**Affiliations:** 1 Sohyaku. Innovative Research Division, Mitsubishi Tanabe Pharma Corporation, 1000 Kamoshida-cho, Aoba-ku, Yokohama, Kanagawa 227-0033 Japan; 2 Center of Experimental Rheumatology, Department of Rheumatology, University Hospital Zurich, University of Zurich, Wagistr. 14, 8952 Schlieren, Switzerland; 3 Leeds Institute of Rheumatic and Musculoskeletal Medicine, Faculty of Medicine and Health, University of Leeds, Leeds, LS9 7TF UK; 4 NIHR Leeds Musculoskeletal Biomedical Research Centre, Leeds, UK; 5 Department of Internal Medicine 3—Rheumatology and Immunology, Friedrich-Alexander-University Erlangen-Nürnberg (FAU) and University Hospital Erlangen, Erlangen, Germany

**Keywords:** Systemic sclerosis, Melanocortin 1 receptor, Fibroblast, Inflammation, Vascular dysfunction, Fibrosis

## Abstract

**Background:**

Activation of melanocortin 1 receptor (MC1R) is known to exert broad anti-inflammatory and anti-fibrotic effects. The purpose of this study is to investigate the potential of dersimelagon, a novel oral MC1R agonist, as a therapeutic agent for systemic sclerosis (SSc).

**Methods:**

The effects of dersimelagon phosphoric acid (MT-7117) on skin fibrosis and lung inflammation were evaluated in bleomycin (BLM)-induced SSc murine models that were optimized for prophylactic and therapeutic evaluation. Microarray-based gene expression analysis and serum protein profiling were performed in the BLM-induced SSc models. The effect of MT-7117 on transforming growth factor-β (TGF-β)-induced activation of human dermal fibroblasts was evaluated in vitro. Immunohistochemical analyses of MC1R expression in the skin of SSc patients were performed.

**Results:**

Prophylactic treatment with MT-7117 (≥ 0.3 mg/kg/day p.o.) significantly inhibited skin fibrosis and lung inflammation, and therapeutic treatment with MT-7117 (≥ 3 mg/kg/day p.o.) significantly suppressed the development of skin fibrosis in the BLM-induced SSc models. Gene array analysis demonstrated that MT-7117 exerts an anti-inflammatory effect via suppression of the activation of inflammatory cells and inflammation-related signals; additionally, vascular dysfunction was extracted as the pathology targeted by MT-7117. Serum protein profiling revealed that multiple SSc-related biomarkers including P-selectin, osteoprotegerin, cystatin C, growth and differentiation factor-15, and S100A9 were suppressed by MT-7117. MT-7117 inhibited the activation of human dermal fibroblasts by suppressing TGF-β-induced *ACTA2* (encoding α-smooth muscle actin) mRNA elevation. MC1R was expressed by monocytes/macrophages, neutrophils, blood vessels (endothelial cells), fibroblasts, and epidermis (keratinocytes) in the skin of SSc patients, suggesting that these MC1R-positive cells could be targets for MT-7117.

**Conclusions:**

MT-7117 demonstrates disease-modifying effects in preclinical models of SSc. Investigations of its mechanism of action and target expression analyses indicate that MT-7117 exerts its positive effect by affecting inflammation, vascular dysfunction, and fibrosis, which are all key pathologies of SSc. The results of the present study suggest that MT-7117 is a potential therapeutic agent for SSc. A phase 2 clinical trial investigating the efficacy and tolerability of MT-7117 in patients with early, progressive diffuse cutaneous SSc is currently in progress.

**Supplementary Information:**

The online version contains supplementary material available at 10.1186/s13075-022-02899-3.

## Background

Systemic sclerosis (SSc; also termed scleroderma) is an autoimmune disease characterized by the following major pathologies: dysregulation of immunity/inflammation, microvascular dysfunction, and generalized fibrosis in multiple organs [1]. Although skin sclerosis is a prominent feature of SSc, the pathological changes in the lungs, gastrointestinal tract, kidneys, and heart determine the clinical outcome. Especially, assessment and treatment of the lung are important because SSc-associated interstitial lung disease (ILD) is the leading cause of death in SSc patients. Although skin involvement is not a cause of mortality per se, substantial skin involvement or rapidly progressive skin involvement early in the disease is associated with increased mortality and prevalence of internal organ involvement [2, 3]. SSc has the highest case-specific mortality among autoimmune rheumatic diseases [4]. In the process of fibrosis, although the site and factors that trigger the initial injury may often vary from organ to organ, the common molecular mechanisms in various organs have been reported to exist [5, 6]. In such cases, pathways and mechanisms in skin fibrosis can be extrapolated to lung fibrosis [7]. Although intensive research over the past few years has improved the understanding of the disease, only two drugs, nintedanib (Ofev®) [8] and tocilizumab (Actemra®) [9, 10], have so far been approved by the US Food and Drug Administration (FDA) for the treatment of SSc-associated ILD. Nevertheless, there are no approved drugs for SSc, other than SSc-associated ILD, and therefore, there is a great unmet medical need for novel therapies that are widely effective against fibrosis of not only the lung but also many organs including the skin in SSc.

The melanocortin receptor (MCR) family belongs to the class A family of G protein-coupled receptors and consists of five members with different tissue distribution and functions: MC1R, MC2R, MC3R, MC4R, and MC5R [11]. The expression of MC1R on various cell types including melanocytes, monocytes, endothelial cells, fibroblasts, and keratinocytes has been reported [12]. It is well known that activation of MC1R by α-melanocyte-stimulating hormone (αMSH), its endogenous ligand, induces melanin production in melanocytes, which results in skin and hair pigmentation [13, 14]. In addition, activation of MC1R is known to exert broad anti-inflammatory effects [15]. In vitro, αMSH-mediated inhibition of activation of nuclear factor-κB, a well-known master regulator of inflammation, has been reported [16]. Extensive studies have demonstrated that αMSH suppresses the production of proinflammatory cytokines—tumor necrosis factor-α (TNF-α), interleukin (IL)-1, IL-6, IL-8, keratinocyte-derived chemokine (KC) and interferon-γ—and expression of adhesion molecules—vascular cell adhesion molecule-1 and E-selectin—on inflammatory and endothelial cells. Moreover, αMSH was reported to be a suppressor of proinflammatory, non-cytokine regulators such as inducible nitric oxide synthase, prostaglandin E2, and reactive oxygen species. Another proposed anti-inflammatory action of αMSH is its induction of IL-10, a well-known, potent suppressor of cytokines in keratinocytes and monocytes [17]. In vivo, αMSH has been reported to show anti-inflammatory effects in the several disease models, such as lipopolysaccharide (LPS)-induced sepsis [18], adjuvant-induced arthritis [19], and dextran sulfate sodium-induced colitis [20] models. αMSH has also been reported to show an anti-vascular dysfunction effect in LPS-induced cutaneous vasculitis [21] and ischemic/reperfused models [22, 23]. In terms of its relationship with SSc, αMSH has been reported to suppress skin fibrosis in the bleomycin (BLM)-induced skin fibrosis model, which is the most widely used SSc animal model [24], and deficient MC1R signaling exacerbates BLM-induced skin fibrosis in mice [25]. These pieces of evidence suggest that MC1R agonism has potential in the treatment of SSc because of its anti-inflammatory, anti-vascular dysfunction, and anti-fibrotic effects. However, the mechanisms, by which MC1R mediates these effects in SSc, remain incompletely understood. Information on the expression level and distribution of MC1R in SSc patients is poorly understood, and only a few reports have identified *MC1R* gene expression in skin biopsy samples or skin fibroblasts from few SSc patients [24, 26].

Dersimelagon phosphoric acid (MT-7117) is an orally bioavailable small molecule synthesized by Mitsubishi Tanabe Pharma Corporation that is a selective agonist for MC1R. MT-7117 behaves as a full agonist for MC1R and is selective for MC1R among other MCRs. Moreover, MT-7117 was shown to darken the coat color of mice and the skin of monkeys by inducing pigmentation in vivo [27]. MT-7117 is currently being studied in a phase 3 clinical trial (NCT04402489) as a potential therapeutic option for patients with a history of phototoxic reactions from erythropoietic protoporphyria or X-linked lymphoproliferative syndromes.

In the present study, we evaluated the efficacy of MT-7117 in preclinical SSc models in vitro and in vivo focusing on the anti-inflammatory and anti-fibrotic effects, which highlights its effects other than those on melanin production. The target organs of efficacy evaluation and mechanism of action analysis of MT-7117 were the lung and skin. Additionally, the expression of MC1R in the skin of SSc patients was investigated in detail by performing immunostaining using a large number of patient samples. Based on these evaluations, we investigated the possibility of MT-7117 as a treatment for SSc. The results presented herein provided the scientific basis for an on-going phase 2 clinical trial (NCT04440592) with MT-7117 in patients with diffuse cutaneous SSc (dcSSc) [28].

## Materials and methods

### Test substance

MT-7117 synthesized at Mitsubishi Tanabe Pharma Corporation was dissolved in dimethylsulfoxide (DMSO) for in vitro assays or suspended in 0.5% methylcellulose solution for in vivo experiments.

### BLM-induced skin fibrosis and lung inflammation—prophylactic evaluation

Ten-week-old female C3H/HeNCrlCrlj mice (Charles River Laboratories Japan, Inc.) were used, and all animal experiments were conducted in accordance with the Guidelines for Animal Experimentation of Mitsubishi Tanabe Pharma Corporation (approval number: BJ14-0699). The backs of mice were shaved, and the middle of the backs was marked with oil-based red ink. Phosphate-buffered saline (PBS) or BLM (Nippon Kayaku, Tokyo, Japan) (0.15 mg/0.1 mL per animal) was subcutaneously injected at the marked site once daily from day 0 to day 25 (since repeated BLM injections reduced the body weights of the mice remarkably, BLM injection was suspended several times for all groups). MT-7117 solutions at 0.1 mL/10 g of body weight were orally administered once daily (approximately 2 h before the BLM injection) for 29 consecutive days from day 0 until the day before the end of the evaluation (day 28). On day 29, mice were euthanized by inhalation anesthesia with isoflurane, and whole blood and back skin samples were collected. The left lung was dissected, weighed, and immersed in RNAlater solution (Qiagen, Hilden, Germany). The collected blood was separated into serum. The injection site of the skin was excised, weighed (wet weight), and immersed in 0.5 mol/L acetic acid solution containing 0.3 mg/mL pepsin to solubilize collagen. The collagen content of the solubilized sample was explored using the QuickZyme Soluble Collagen Assay kit (QuickZyme Biosciences, Leiden, Netherlands), and absorbance was determined by a microplate reader (VERSAmax, Molecular Devices, Sunnyvale, CA, USA). Serum levels of surfactant protein D (SP-D) were assayed using the Rat/Mouse SP-D kit YAMASA EIA (Yamasa Corporation, Choshi, Japan), and absorbance was determined by a microplate reader (VERSAmax). Total RNA of lungs was isolated using RNeasy 96 Universal Tissue Kit (Qiagen) according to the manufacturer’s instructions. Real-time PCR was performed using the One Step SYBR PrimeScript PLUS RT-PCR Kit (Takara Bio Inc., Kusatsu, Japan) and ABI PRISM 7900HT Real-Time PCR System (Applied Biosystems, Foster City, CA, USA). Takara perfect real-time primer pairs (Takara Bio Inc.) were used to analyze the gene expression of chemokine CC ligand-2 (*Ccl2*, also known as monocyte chemotactic protein-1 (*Mcp-1*), MA066003), interleukin-6 (*Il-6*, MA039013), and hypoxanthine phosphoribosyltransferase (*Hprt*, MA031262). Quantification was performed using the comparative CT (cycle threshold) method employing *Hprt* as the housekeeping gene.

### Pharmacokinetic study of MT-7117

A pharmacokinetic study of MT-7117 was performed using the BLM-induced SSc model (prophylactic evaluation) described above. After repeated oral administrations of MT-7117 once daily for 29 days, whole blood was collected from individual mice at each sampling point (1, 3, 6, and 24 h after the last dose) and serum was separated from blood. Serum concentration of MT-7117 was measured by an API 5000 MS/MS system (AB Sciex) coupled with Agilent 1200 HPLC (Agilent Technologies, Santa Clara, CA, USA).

### Pre-established BLM-induced skin fibrosis—therapeutic evaluation

Evaluation of the therapeutic effect of MT-7117 using a pre-established BLM-induced skin fibrosis model was performed as previously described [29] and is briefly described as follows. Six to 7-week-old female C57BL/6 mice (Laboratory Animal Service Center, University of Zurich) were used, and all animal experiments were conducted in accordance with the European Community Council Directive for Care and Use of Laboratory Animals and the Swiss law (approval number: ZH002/17). BLM (Baxter AG, Deerfield, IL, USA) was dissolved in saline and subcutaneously administered every other day (0.1 mg/0.1 mL per animal) in defined areas of the upper back for 6 weeks (day 1 to 42). During the last 3 weeks of the experimental period (day 22 to 42), the mice were orally administered MT-7117, imatinib (Selleckchem, Huston, TX, USA), or vehicle once daily. On day 43, the mice were euthanized by CO_2_ inhalation, and back skin and serum samples were collected.

Mouse skin samples were fixed in 4% formalin and embedded in paraffin. Hematoxylin and eosin (HE) staining was performed following standard procedures. Skin thickness was determined by measuring the thickness of HE-stained skin samples using a microscope with an image analysis program (Zeiss Imager Z1, Carl Zeiss AG, Oberkochen, Germany). Skin thickness was measured by randomly selecting 3 records of non-overlapping pictures taken at 100× magnification of each piece of HE-stained mouse skin sample (in total, 6 pictures and 18 measurements per sample). Analyses were performed by two independent examiners in a blinded manner. Each value of skin thickness was calculated as an average of 36 measurements.

α-smooth muscle actin (αSMA, encoded by *ACTA2*) staining was performed for skin sections. After deparaffinization and rehydration, 10% goat serum (Vector Laboratories, Burlingame, CA, USA) was used to prevent unspecific antibody binding. The samples were incubated with primary antibody (1:750, monoclonal mouse anti-αSMA, clone 1A4, Sigma-Aldrich, St. Louis, MO, USA) for 1 h at room temperature, followed by incubation with alkaline phosphatase-labeled goat anti-mouse secondary antibody (Dako, Glostrup, Denmark) for 30 min at room temperature. The numbers of αSMA-positive myofibroblasts in the skin were determined manually using a microscope with an image analysis program (Zeiss Imager Z1). Quantification of αSMA-positive myofibroblasts in mouse skin was performed for 6 slides by recording 6 randomly selected, non-overlapping areas (172.5 × 218 μm, 37605 μm^2^) per slide of mouse skin at 400× magnification. Two independent examiners performed the analyses in a blinded manner. The number of αSMA-positive myofibroblasts in an area of 37605 μm^2^ was calculated as an average of 12 values.

Masson’s trichrome staining of skin sections was performed following standard procedures. Masson’s trichrome-stained sections were observed using a microscope with an image analysis program (Zeiss Imager Z1) at 100× magnification.

### Microarray-based gene expression analysis

#### Gene expression profiling with Agilent Expression Array

Total RNA of mouse lung tissue samples of PBS_vehicle (control group without disease), BLM_vehicle (control group with disease), and BLM_MT-7117 (0.3 mg/kg) obtained from the BLM-induced SSc model (prophylactic evaluation) was used for analysis. The purity, concentration, and quality of the RNA samples were confirmed with NanoDrop 2000 (Thermo Fisher Scientific, Waltham, MA, USA) and Agilent 2100 bioanalyzer (Agilent Technologies). The RNA was then Cy3-labeled using a Low Input Quick Amp Labeling Kit, one-color (Agilent Technologies). Labeled cRNA samples were fragmented and then hybridized with the Agilent SurePrint G3 Mouse GE v2 8 × 60 K Microarray (Agilent Technologies) using a Gene Expression Hybridization Kit (Agilent Technologies). After hybridization, the microarray was washed with the Gene Expression Wash Buffers Pack (Agilent Technologies) and imaged with a scanner to detect the signals. Signal intensities were evaluated with Agilent Feature Extraction software 12.0.

#### Bioinformatics analysis

Bioinformatics analysis in this study was performed with software, including GeneSpring GX 14.9.1, Ingenuity Pathways Analysis (IPA) 2019 autumn, and Microsoft Excel 2010. Genes that fluctuated ≥ 2 fold in the BLM_vehicle group in comparison with those of the PBS_vehicle group and that satisfied a *p*-value < 0.05 of the moderated *t*-test were defined as “differentially expressed genes (DEGs) in the disease model.” Among DEGs, the genes in the MT-7117 treatment group that fluctuated ≥ 1.5 fold in the opposite direction and met a *p*-value < 0.05 of the moderated *t*-test in comparison with those of the BLM_vehicle group were defined as “DEGs by MT-7117 treatment.” We inputted DEGs into IPA and performed two types of calculations: “Canonical pathways” and “Diseases or functions.” In this report, “Canonical pathways” is described as “pathways” and “Diseases or functions” as “category.” The former contains a list for a series of gene interactions and the latter is a list related to diseases and biological functions. These datasets are registered in the IPA software. Initially, Fisher’s exact test was used to calculate the probability of overlap between two sets of genes. Thereafter, downstream effect analysis (DSEA) [30] was used to calculate the activation *z*-score. DSEA is a technique for predicting the direction of change in expression patterns (either activation or inhibition) based on the expected causal effects between genes and functions. Based on the results of the DSEA analysis, information about the cell type (macrophages, neutrophils, mononuclear leukocytes, T-lymphocytes, B-lymphocytes, smooth muscle cells, endothelial cells, epithelial cells, and fibroblasts) that may be involved in the pathogenesis of SSc was extracted. Subsequently, we extracted information regarding biological functions and molecular signaling pathways, such as inflammation/immune abnormality, vascular dysfunction, and fibrosis, involved in the pathogenesis of SSc.

### Serum protein profiling

Serum samples were obtained from mice of the BLM-induced SSc model (therapeutic evaluation). Samples from the saline_vehicle (control group without disease), BLM_vehicle (control group with disease), BLM_MT-7117 (10 mg/kg), and BLM_imatinib (150 mg/kg) groups were used for analysis. Using Luminex® assays (R&D systems, Minneapolis, MN, USA), 110 proteins were investigated (supplementary Table S1). Proteins satisfying any of the following criteria were analyzed: (i) all samples were adequately measured within the quantitative range; (ii) more than half of the samples in one or more groups were quantified, although some samples were out of the quantitative range. The values of samples that were out of the quantitative range were substituted by those within the lower and upper limits.

### Skin biopsy samples and fibroblast isolation (fibroblast assay)

Full-thickness skin biopsies were obtained from clinically involved skin of patients affected by dcSSc, who were recruited from the Scleroderma clinic within the Leeds Institute of Rheumatic and Musculoskeletal Medicine (UK). Control skin samples were from healthy donors. All patients with dcSSc and healthy donors provided written informed consent through the stratification for risk of progression in scleroderma (STRIKE SSC), which had been approved by the North–East Research Ethics committee (number 15/NE/0211). Patients with SSc fulfilled the 2013 ACR/EULAR classification criteria for SSc [31] and were classified as having dcSSc according to the LeRoy and Medsger criteria [32].

Dermal fibroblasts were isolated from the skin biopsy samples of healthy donors and SSc patients. The skin biopsy samples were minced with a scalpel, placed in plastic culture dishes, and covered with Dulbecco’s modified Eagle medium (DMEM) with 4.5g/L glucose (Thermo Fisher Scientific) supplemented with 20% fetal calf serum (FCS, Thermo Fisher Scientific), 1% penicillin and streptomycin (Merck, Darmstadt, Germany), and 1 μg/ml of amphotericin (Thermo Fisher Scientific) and cultured at 37°C in a humidified atmosphere of 5% CO_2_. Additional growth medium without amphotericin was added after 3 days. After 7 days, the culture medium was replaced with a medium containing 10% FCS and subsequently replenished every 2 to 3 days. When a visible outgrowth of cells was obtained, the fibroblasts were passaged. Briefly, cells were incubated with PBS containing 0.1% ethylene diamine tetra-acetic acid (EDTA) for 5 min and then detached with trypsin–EDTA solution (Merck) and cultured until 70–80% confluent in DMEM (1 g/L glucose) supplemented with 10% FCS and 1% penicillin and streptomycin. After a subsequent passage, primary cells were retrovirally immortalized using human telomerase reverse transcriptase as previously described [33].

### Treatment of fibroblasts

Cells were grown to confluence in six-well culture plates, then serum starved in DMEM 1% FCS for 24 h, followed by a 24-h incubation in the presence or absence of transforming growth factor-β1 (TGF-β1, 10 ng/mL) (Sigma-Aldrich). MT-7117 or αMSH (Peptide Institute, Osaka, Japan) was added to the culture medium during TGF-β1 stimulation. DMSO (0.1%) was added to all non-MT-7117-containing wells to normalize for the DMSO-induced effects on cells.

### RNA isolation and real-time PCR analysis (fibroblast assay)

Total RNA was extracted using the Zymo quick RNA mini prep kit according to the manufacturer’s instructions (Zymo Research Corporation, Irvine, CA, USA). First-strand cDNA was synthesized using the High-Capacity cDNA Reverse Transcription kit (Thermo Fisher Scientific). Quantitative RT-PCR was performed in triplicates using SYBR Green RT-PCR Mastermix Kit (Thermo Fisher Scientific) and the ABI PRISM 7500 Fast Real-Time PCR System (Applied Biosystems). Quantification was performed using the comparative CT method employing glyceraldehyde-3-phosphate dehydrogenase (*GAPDH*) as the housekeeping gene. Primer sequences used in qPCR analyses were as follows: *GAPDH* forward 5′-ACC CAC TCC TCC ACC ACC TTT GA-3′, reverse 5′-CTG TTG CTG TAG CCA AAT TCG T-3′; *ACTA2* forward 5′-TGT ATG TGG CTA TCC AGG CG-3′, reverse 5′-AGA GTC CAG CAC GAT GCC AG-3′; and collagen type I alpha 1 (*COL1A1*) forward 5′-GCT CCG ACC CTG CCG ATG TG-3′, reverse 5′-CAT CAG GCG CAG GAA GGT CAG C-3′.

### Skin biopsy samples (immunohistochemical analysis of MC1R)

Full-thickness skin biopsies were obtained from the clinically involved skin of patients affected by dcSSc (*n* = 50) or limited cutaneous SSc (lcSSc, *n* = 10) who were recruited from the University of Erlangen–Nuremberg (Germany). Control skin samples (*n* = 30) were from healthy donors. All patients with SSc and healthy donors provided written informed consent as approved by the institutional ethics committee. Patients with SSc fulfilled the 2013 ACR/EULAR classification criteria for SSc [31] and were classified as having dcSSc according to the LeRoy and Medsger criteria [32]. The study was approved by the ethical review board of the Medical Faculty of the University of Erlangen–Nuremberg (number 3766).

### Immunohistochemical analyses of MC1R

Immunohistochemical analysis of paraffin-embedded sections was performed as previously described [34, 35].

#### Single staining

Protein expression of MC1R in skin biopsy samples was detected by incubation with an anti-MC1R monoclonal antibody (1:50, clone EPR6530; Abcam, Cambridge, UK). Peroxidase-labeled goat anti-rabbit secondary antibody (Dako) was used as the secondary antibody. Isotype-matched antibodies were used as controls. Staining was visualized with 3,3′-diaminobenzidine (Sigma-Aldrich) and peroxidase substrate solution (Sigma-Aldrich). A nuclear counterstain was performed using Meyer’s hematoxylin (J.T. Baker, Phillipsburg, NJ, USA). For permanent mounting, the sections were dehydrated and dried, followed by mounting using Pertex (Histolab, Gothenburg, Sweden). A qualitative assessment was performed by assigning a score based on staining intensity after identifying each stained cell type and tissue element (the number of positive cells was not taken into account for the scoring). The staining intensity scale used for the evaluation is as follows: no staining was scored as 0, faint staining was scored 0.5, light staining was scored as 1, moderate staining was scored as 2, and dark staining was scored as 3.

#### Double staining

To identify the cell types in the skin that were positive for MC1R, double staining for cell types of interest was performed on nine or ten samples randomly selected from those positive for single MC1R staining. The anti-MC1R monoclonal antibody was reacted in the same manner as described above. Alexa Fluor 488-labeled donkey anti-rabbit secondary antibody (Life Technologies, Carlsbad, CA, USA) was used as the secondary antibody. Subsequently, double staining was performed using the following antibodies against cell-specific markers: anti-prolyl-4-hydroxylase β antibodies (1:50, Acris Antibodies, Herford, Germany) for fibroblasts, anti-CD68 antibodies (1:200, Biolegend, San Diego, CA) for monocytes/macrophages, anti-CD66b antibodies (1:250, Biolegend) for neutrophils, and anti-CD31 antibodies (1:50, R&D Systems) for endothelial cells. Antibodies labeled with Alexa Fluor 594 (Invitrogen, Carlsbad, CA, USA) were used as secondary antibodies. For nuclear counterstaining, 4′,6′-diamino-2-phenylindole (Sigma-Aldrich) was used. Immunofluorescence-stained tissue sections were analyzed using a Nikon Eclipse 80i microscope (Nikon, Tokyo, Japan).

### Statistical analysis

All quantitative pharmacological data were expressed as mean ± standard error of the mean (SEM). Statistical differences between groups in each study were assessed as described below. All analyses were performed using the SAS system, and tests were two-tailed with a significance level of < 0.05 or one-tailed with a significance level of < 0.025.

#### BLM-induced SSc murine model—prophylactic evaluation

Differences between PBS_vehicle (control group without disease) and BLM_vehicle (control group with disease) groups were analyzed using Student’s *t*-test. Differences between the BLM_vehicle and MT-7117-treated groups were analyzed using Williams’ test. According to repeated measures analysis of variance (group effect and interaction effect; group × time point), body weights were significantly different (*p* < 0.01) in the groups mentioned above during the 29-day course of the experiment. Therefore, differences in body weight at each time point were analyzed between the PBS_vehicle and BLM_vehicle groups using Student’s *t-*test. Differences between the BLM_vehicle and MT-7117-treated groups were analyzed using Williams’ test.

#### BLM-induced SSc murine model—therapeutic evaluation

Differences between the saline_vehicle (control group without disease) and BLM_vehicle (control group with disease) groups and between the BLM_vehicle and imatinib-treated groups were analyzed using the Wilcoxon test (two-sided). Differences between the BLM_vehicle and MT-7117-treated groups were analyzed using Shirley–Williams’ multiple comparison test (one-sided).

#### Analysis of serum protein profiling

Differences between the saline_vehicle and BLM_vehicle groups, between the BLM_vehicle and MT-7117-treated groups, and between the BLM_vehicle and imatinib-treated groups were analyzed using Student’s *t*-test.

## Results

### Effects of MT-7117 on BLM-induced skin fibrosis and lung inflammation—prophylactic evaluation

**Fig. 1:**
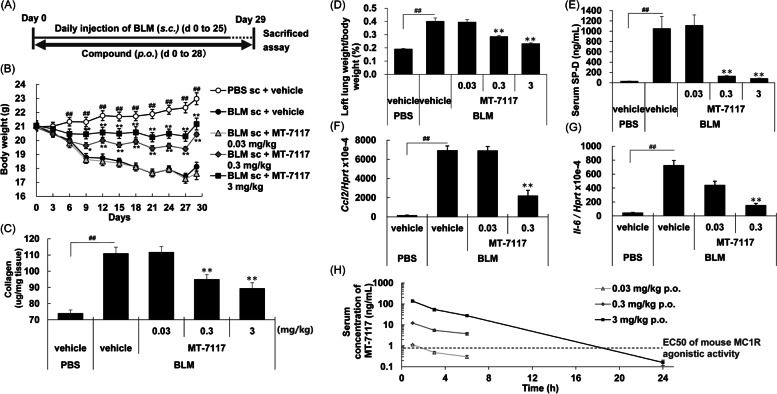
Effects of MT-7117 on skin fibrosis and lung inflammation in the bleomycin (BLM)-induced systemic sclerosis (SSc) model—prophylactic evaluation. BLM was injected daily into the same region of the back skin of mice from day 0 to 25. Mice received oral administration of MT-7117 or vehicle once daily for 29 consecutive days (day 0 to 28). Each sample was collected on day 29. **A** Experimental schedule. **B** Body weight of mice was measured during the experimental period. **C** Collagen content of the skin was measured. **D** Wet weight of the left lung was assessed. **E** Serum surfactant protein D (SP-D) was measured. **F**, **G** Expression of chemokine CC ligand-2 (*Ccl2/Mcp-1*) and interleukin-6 (*Il-6*) mRNA in the lung was determined relative to hypoxanthine phosphoribosyltransferase 1 (*Hprt*). **B**–**E** Each column represents the mean ± SEM (*n* = 10 for all groups, except for the MT-7117-treated group at 0.03 mg/kg, where *n* = 9). ^##^*p* < 0.01 for BLM vs. PBS using Student’s *t*-test; **p < 0.005 for BLM vs. MT-7117 using Williams’ test. **H** Serum concentrations of MT-7117 after 29 days of repeated oral administrations in this model. Each symbol represents the mean ± SD (*n* = 3 at each time point). The dotted line indicates the EC50 of mouse MC1R agonistic activity

The effects of prophylactic treatment with MT-7117 were evaluated using the BLM-induced skin fibrosis and lung inflammation model. We found that daily subcutaneous injection of high-dose BLM induced not only skin fibrosis but also lung inflammation and optimized the experimental conditions for simultaneous evaluation of both endpoints as follows: BLM (0.15 mg/animal) was subcutaneously injected once daily from day 0 to day 25, MT-7117 was orally administered once daily from day 0 to day 28, and subsequently, the endpoints were measured on day 29 (Fig. 1A). Subcutaneous injection of BLM decreased the body weights of the mice from day 0 to day 29, which was significantly improved by MT-7117 at ≥ 0.3 mg/kg (Fig. 1B). Subcutaneous injection of BLM significantly increased the collagen content of the skin, indicating the progression of skin fibrosis (Fig. 1C). Similarly, BLM injection significantly increased the serum level of SP-D (lung injury marker secreted by alveolar epithelial type II cells [36]) and the wet weight of the left lung and *Ccl-2*/*Mcp-1* and *Il-6* mRNA expression in the lung, indicating the progression of lung inflammation (Fig. 1D to G). These increases were inhibited significantly by MT-7117 at ≥ 0.3 mg/kg (Fig. 1C to G). These results showed that prophylactic treatment with MT-7117 inhibited skin fibrosis and lung inflammation in a dose-dependent manner at the same dose range. Additionally, we confirmed the serum concentrations of MT-7117 in this model. After 29 days of repeated oral administration of MT-7117 in this mouse model, the serum concentrations of MT-7117 increased in a dose-proportional manner with *C*_max_ of 12.14 ng/mL (= 17.97 nmol/L) at 0.3 mg/kg (Fig. 1H). MT-7117 showed in vitro agonistic activity for mouse MC1R with EC_50_ values of 0.77 ng/mL (= 1.14 nmol/L) [27]. These results indicate that the level of systemic exposure of MT-7117 reasonably exceeded the EC_50_ values of mouse MC1R agonistic activity.

### Effects of MT-7117 on pre-established BLM-induced skin fibrosis—therapeutic evaluation

**Fig. 2:**
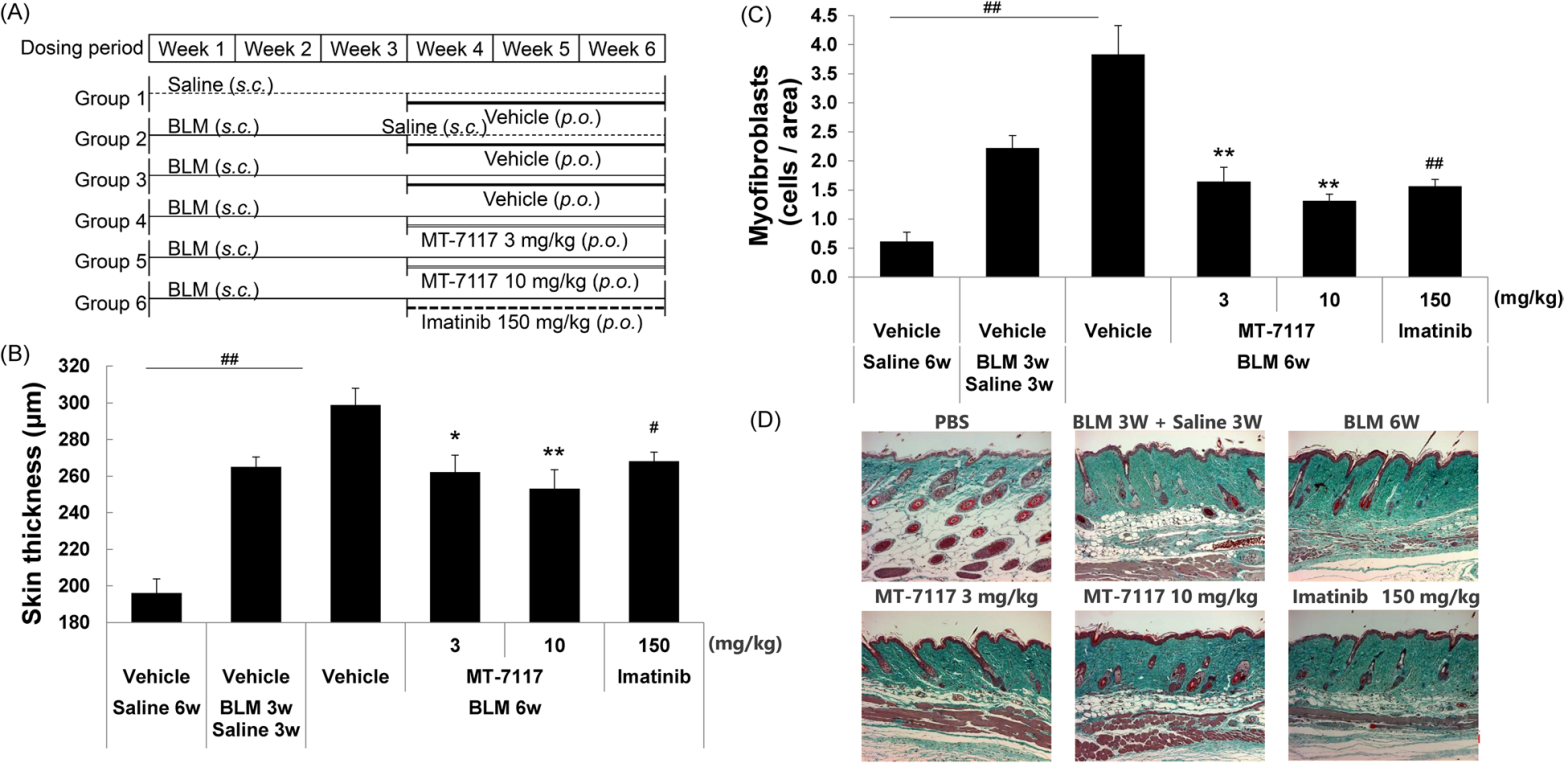
Effects of MT-7117 on skin fibrosis in the pre-established BLM-induced SSc model—therapeutic evaluation. BLM was injected into the back skin of mice every other day for 6 weeks. During the last 3 weeks of the experimental period, the mice received oral administration of MT-7117, imatinib, or vehicle once daily. Skin samples were obtained on day 43 and embedded in paraffin. **A** Experimental schedule. **B** Skin thickness was determined by measuring the thickness of HE-stained skin. **C** α-smooth muscle actin (αSMA, encoded by *Acta2*) staining was performed using paraffin-embedded skin. The numbers of αSMA-positive myofibroblasts in a specific area of skin were determined. **D** Representative image of Masson’s trichrome staining of dorsal skin in each group. **B**, **C** Each column represents the mean ± SEM (*n* = 8). ^#^*p* < 0.05, ^##^*p* < 0.01 for group 3 vs. group 1 and group 3 vs. group 6 using Wilcoxon test (two-sided), *p < 0.025, **p < 0.005 for group 3 vs. group 4 and 5 using Shirley–Williams’ test (one-sided)

This study aimed to evaluate the therapeutic effect of MT-7117 using the BLM-induced skin fibrosis model when MT-7117 was dosed after skin fibrosis had been partially established in mice. Skin fibrosis was gradually induced over 6 weeks under mild conditions by injecting a lower dose of BLM every other day compared with the above-mentioned BLM model for prophylactic evaluation. BLM (0.1 mg/animal) was subcutaneously injected every other day for 6 weeks, and during the last 3 weeks of the experimental period, MT-7117, imatinib, or vehicle was orally administered once daily. Group 2, in which BLM was injected for 3 weeks followed by saline injection for 3 weeks, was set as the group reflecting the fibrosis state at the start of drug administration (Fig. 2A). Subcutaneous injections of BLM for 6 weeks significantly increased skin thickness (Fig. 2B) and the number of αSMA-positive myofibroblasts (αSMA is known as fibroblast activation and myofibroblast transdifferentiation marker [37]) in the dermis (Fig. 2C). Using Masson’s trichrome staining, accumulation of dense collagen fibers in the dermis and loss of dermal white adipose tissue after BLM injection were observed (Fig. 2D). Prolonged injection of BLM for 6 weeks tended to increase the severity of skin fibrosis compared with injections for 3 weeks followed by injections of saline for an additional 3 weeks. The BLM-induced increase of skin thickness and αSMA-positive myofibroblasts was significantly inhibited by MT-7117 at 3 and 10 mg/kg (Fig. 2B, C). MT-7117 ameliorated BLM-induced loss of dermal white adipose tissue (Fig. 2D). Similarly, imatinib, a tyrosine kinase inhibitor used as a positive control [38], at 150 mg/kg also improved these parameters. The results of these parameters in the MT-7117-treated group were almost the same as those in group 2 that were treated with BLM for 3 weeks and saline for 3 weeks, indicating that MT-7117 almost completely inhibited the progression of fibrosis after 3 weeks (Fig. 2B to D). These results demonstrate that MT-7117 suppresses the development of skin fibrosis even if drug intervention is performed after the fibrosis has already been partially induced, but it is unlikely to reverse pre-existing fibrosis. Doses lower than 3 mg/kg were not tested in this model. The minimum effective dose should be determined in future studies.

### Analysis of the mechanism of action of MT-7117 by microarray-based gene expression analysis

**Fig. 3:**
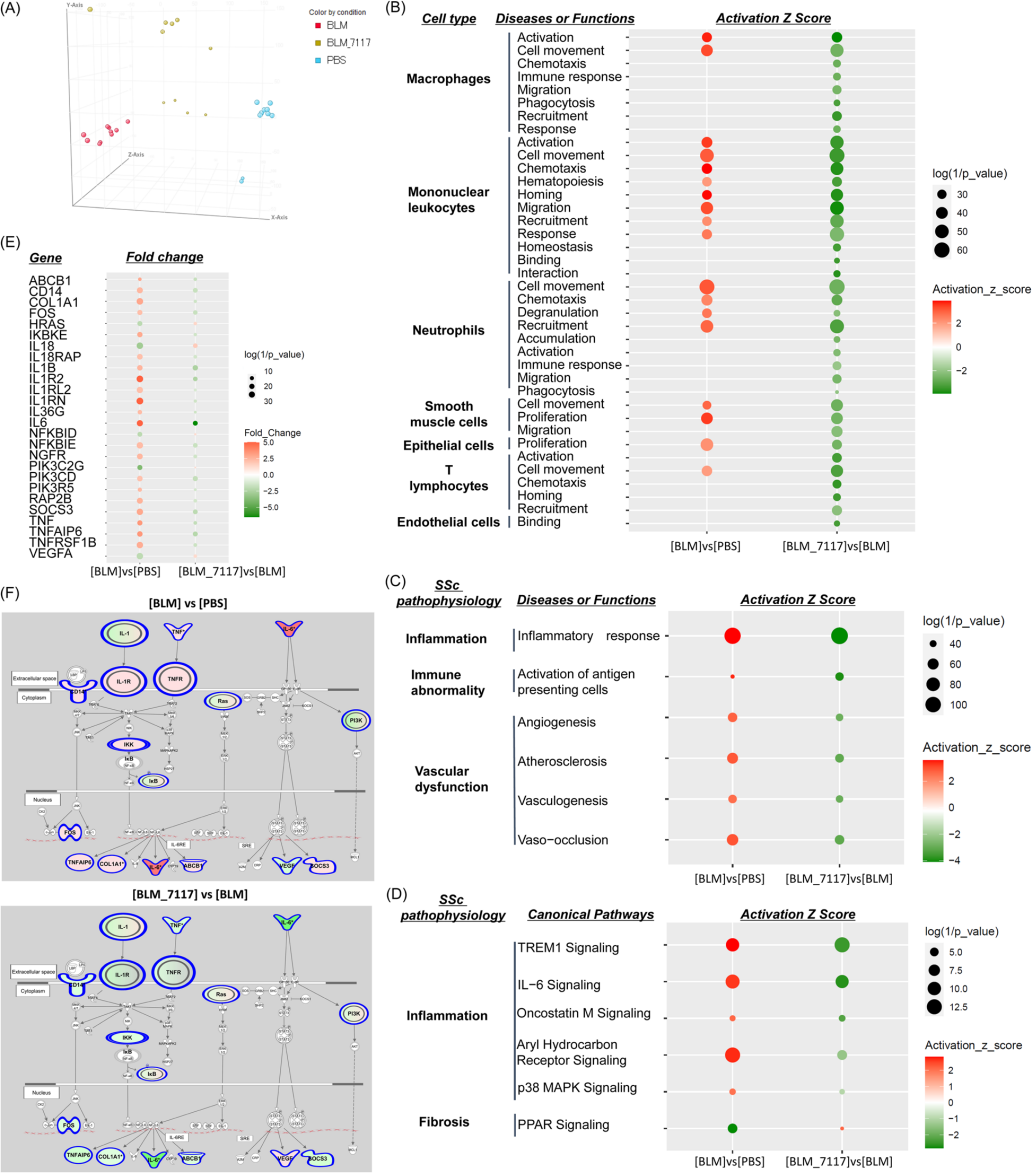
Microarray-based gene expression profiling of lung tissue in the BLM-induced SSc model treated with MT-7117. Lung samples treated with MT-7117 at 0.3 mg/kg from the BLM-induced SSc model (prophylactic model) were used for DNA microarray analysis. Bioinformatics analysis was performed using IPA software, and two types of calculations of two data sets were performed: “Canonical pathways” and “Diseases or functions.” Downstream effect analysis was used to calculate the activation *z*-score. **A** Principal component analysis of gene expression profiles in PBS_vehicle vs. BLM_vehicle vs. BLM_MT-7117 groups. **B** Categories by cell types that changed with BLM and MT-7117 treatment. Categories (**C**) and signaling pathways (**D**) related to SSc pathophysiology that changed with BLM and MT-7117 treatment. The color of symbols represents activation (red) or inactivation (green), and the size reflects the *p-*value. **E** Fluctuating genes involved in IL-6 signaling. **F** Pathway map of IL-6 signaling: fluctuations in PBS vs. BLM groups (left) and BLM vs. MT-7117 groups (right). Red: increased expression; green: decreased expression; solid line: direct relation; dotted line: indirect relation

To explore the mechanism of action of MT-7117, a microarray-based gene expression analysis was performed. Data for DNA microarray analyses of mouse lung tissue samples were obtained from the BLM-induced SSc model (prophylactic evaluation). The number of DEGs in the disease model was 3337, and the number of DEGs by MT-7117 treatment was 1477. The principal component plot showed that PBS_vehicle, BLM_vehicle, and MT-7117-treated groups were clearly separated into different clusters (Fig. 3A).

The results of the analysis using IPA software are described below (the raw data used to prepare Fig. 3 are listed in supplementary Table S2). In the analysis that focused on the cell types related to SSc pathology, the categories that changed in the BLM_vehicle or MT-7117-treated groups where the absolute value of the *z*-score was ≥ 2 are listed in Fig. 3B. Numerous categories related to macrophages, mononuclear leukocytes (monocytes), and neutrophils were extracted, followed by endothelial cell-related categories, suggesting that these cells are main target cells of MT-7117 in the BLM-induced SSc model. T-lymphocyte- and endothelial cell-related categories were not changed by BLM but were suppressed by MT-7117, and none of B-lymphocyte- and fibroblast-related categories met the extraction criteria. In the analysis that focused on biological functions, categories of the inflammatory response (related to inflammation), activation of antigen-presenting cells (related to immune abnormality), angiogenesis, atherosclerosis, vasculogenesis, and vaso-occlusion (related to vascular dysfunction) were activated by BLM and were suppressed by MT-7117 (Fig. 3C). In the analysis that focused on molecular signaling pathways, treatment with MT-7117 resulted in suppression of inflammatory molecular signaling pathways including triggering receptor expressed on myeloid cells-1 (TREM1), IL-6, and oncostatin M, and activation of the peroxisome proliferator-activated receptor (PPAR) signaling pathway associated with fibrosis (Fig. 3D).

With respect to IL-6 signaling, the changes in gene expression of each gene constituting the IL-6 signal pathway are shown in Fig. 3E and are visualized on the pathway map (Fig. 3F). MT-7117 reversed the gene expression fluctuation induced by BLM, regardless of whether the related genes were upstream (involved in the expression of IL-6) or downstream (genes responding to IL-6) in the IL-6 pathway (Fig. 3E, F). Among the component genes of the IL-6 signaling pathway, the expression of the IL-6 gene itself was remarkably upregulated by BLM and was downregulated the most by MT-7117 (Fig. 3E, F).

### Analysis of serum biomarkers of MT-7117

**Fig. 4:**
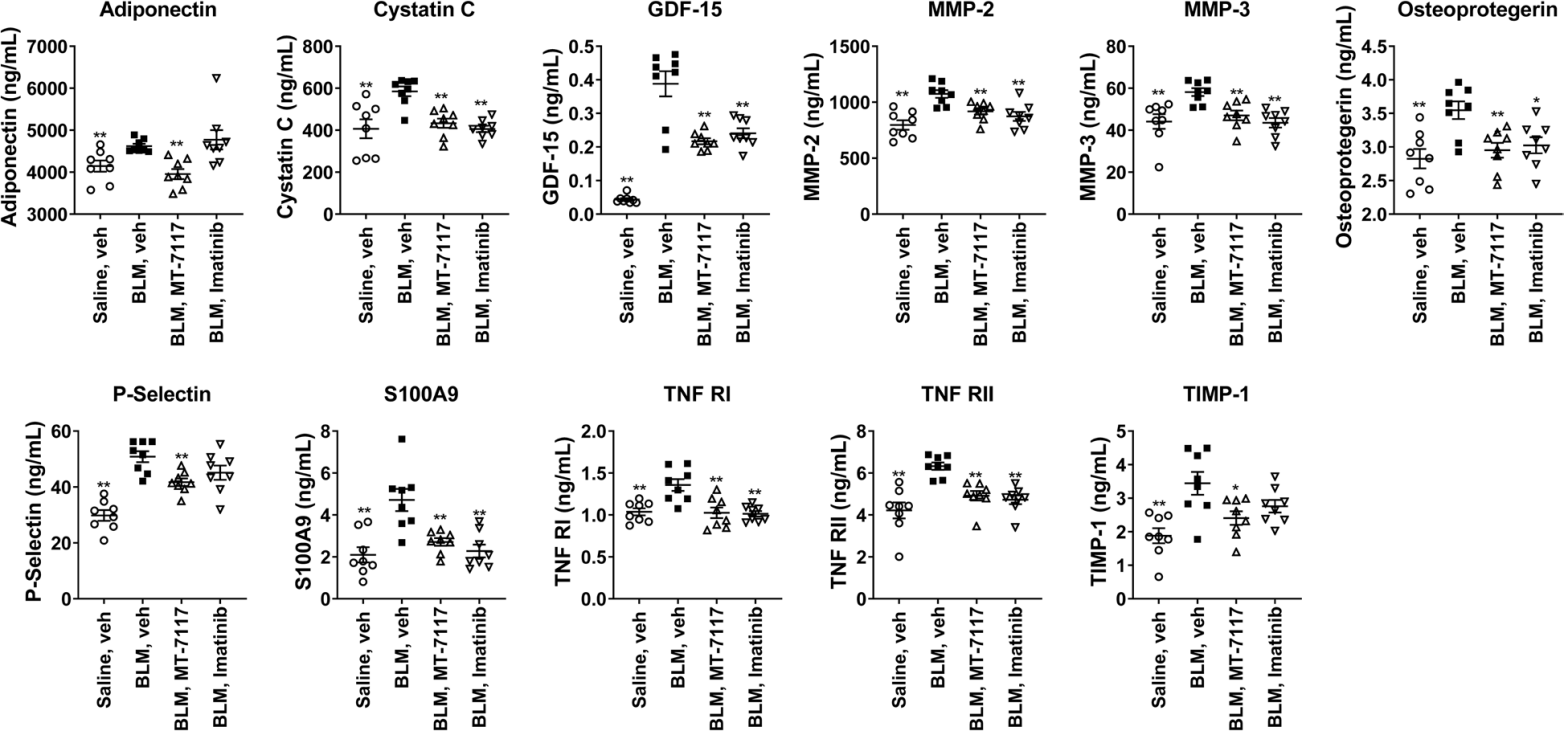
Serum protein profiling in the BLM-induced SSc model treated with MT-7117. Serum samples from mice treated with MT-7117 at 10 mg/kg and imatinib at 150 mg/kg from the BLM-induced SSc model (therapeutic model) were used for serum protein profiling. Using Luminex® assays, 110 proteins were investigated. The graphs showed 11 proteins that were significantly suppressed by MT-7117 treatment. All values are expressed as an individual plot dot and the mean ± SEM (*n* = 8). *p < 0.05, **p < 0.01 by Student’s *t*-test (vs. BLM_vehicle group)

To explore serum biomarkers of MT-7117, serum protein profiling was performed using serum samples obtained from the BLM-induced SSc model (therapeutic evaluation). A total of 110 serum protein profiles were investigated by Luminex® assays (the measured factors are listed in supplementary materials, Table s1). Of the 110 proteins measured, 67 met the criteria for analysis as described in the Material and Methods (the results of 67 proteins are shown in supplementary Fig. s1). The concentrations of adiponectin, cystatin C, growth and differentiation factor-15 (GDF-15), matrix metalloproteinase (MMP)-2, MMP-3, osteoprotegerin, P-selectin, S100A9, tumor necrosis factor receptor (TNFR) I, TNFRII, and tissue inhibitor of metalloproteinases-1 (TIMP-1) were significantly elevated by BLM, and MT-7117 significantly suppressed these elevations (Fig. 4).

### Effect of MT-7117 on human fibroblast activation

**Fig. 5:**
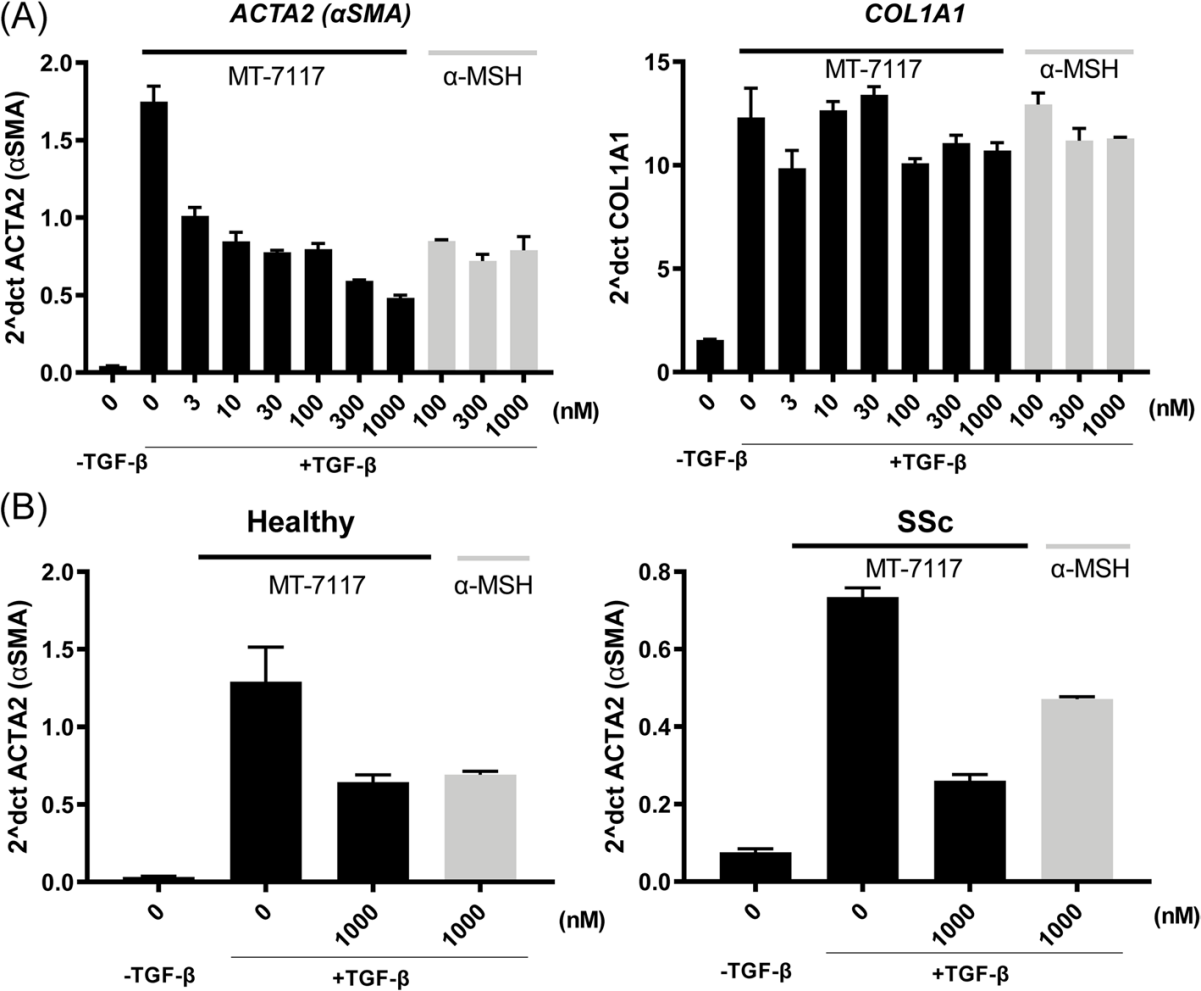
Effect of MT-7117 on human fibroblast activation. Healthy or SSc dermal fibroblasts were grown to confluence and then starved in a medium containing 1% fetal calf serum for 24 h. Afterwards, cells were stimulated with TGF-β (10 ng/mL) in the presence of MT-7117 or αMSH for 24 hours. **A** *ACTA2* and *COL1A1* mRNA expression in healthy dermal fibroblasts was determined by real-time PCR and normalized by *GAPDH* expression during a dose-response treatment of both compounds. **B** *ACTA2* and *COL1A1* mRNA expression in healthy and SSc dermal fibroblasts were determined by real-time PCR and normalized by *GAPDH* expression using 1000 nmol/L of both compounds. Each column represents the mean ± SEM of triplicate samples. Results are representative of 2 to 3 independent experiments with similar findings

To assess the anti-fibrotic effect of MT-7117 on skin fibroblasts, we next evaluated the effects of MT-7117 on *ACTA2* and *COL1A1* mRNA expression induced by TGF-β stimulation. After 24-h culture in TGF-β-containing medium following 24 h of starvation, a marked increase in *ACTA2* and *COL1A1* mRNA expression was demonstrated in healthy dermal fibroblasts. MT-7117 inhibited the TGF-β-induced *ACTA2* mRNA elevation in a concentration-dependent manner but had no effect on *COL1A1* mRNA expression. A similar tendency was observed with treatment using αMSH, an endogenous ligand for MC1R (Fig. 5A). Furthermore, we evaluated the effects of MT-7117 and αMSH on dermal fibroblasts derived from patients with SSc. MT-7117 and αMSH suppressed the TGF-β-induced increase in *ACTA2* mRNA expression at a concentration of 1000 nmol/L in fibroblasts from SSc patients as well as fibroblasts from healthy donors (Fig. 5B). These results suggest that MT-7117 has an anti-fibrotic effect by suppressing TGF-β-induced fibroblast activation.

### Analysis of MT-7117 targets by immunostaining of MC1R in the human skin

**Fig. 6:**
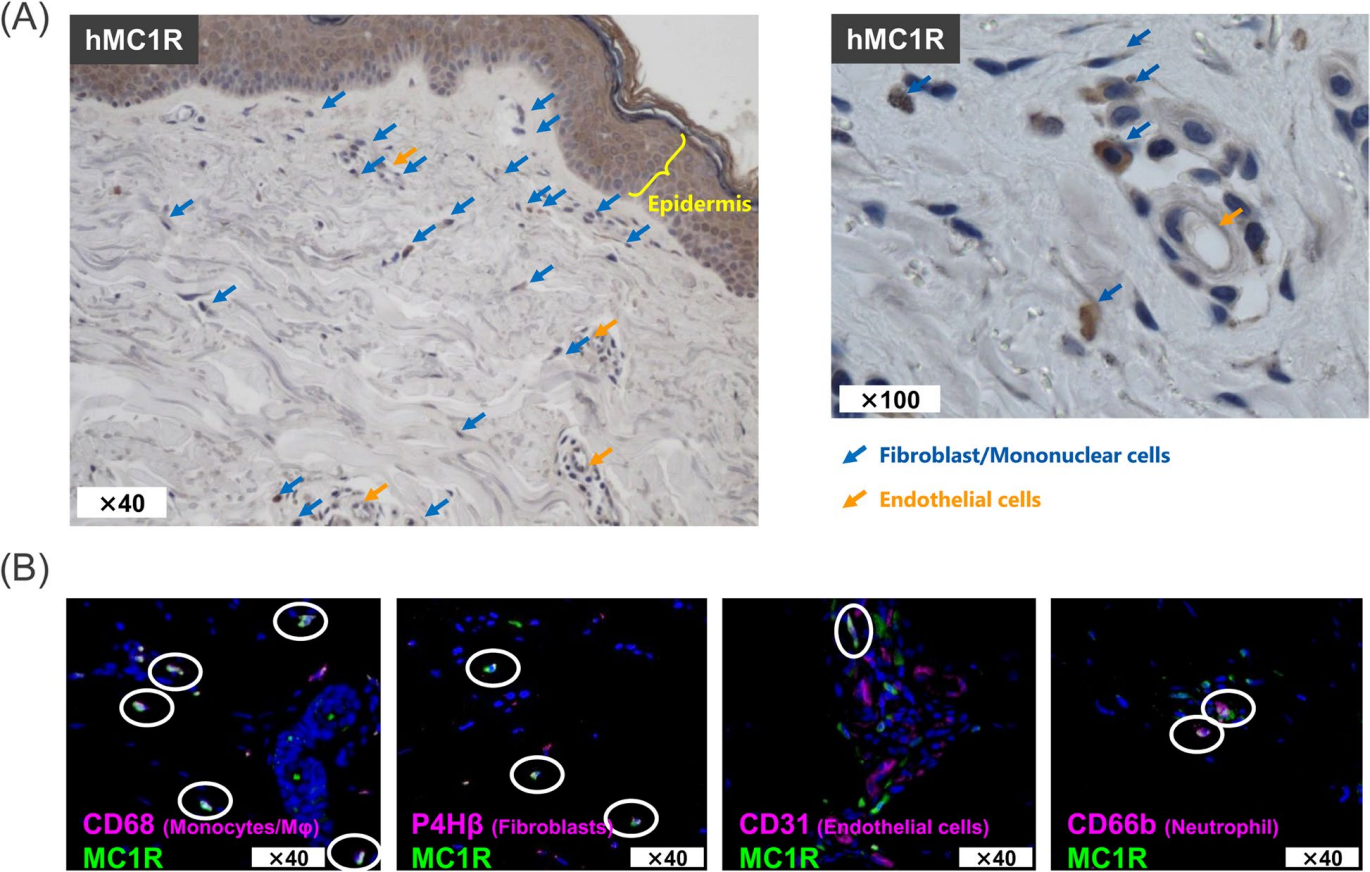
Immunohistochemical analysis of MC1R in skin biopsies from patients with SSc. Representative images of immunohistochemical staining of skin biopsy from dcSSc patients. **A** Single staining with anti-MC1R antibody visualized by DAB substrate (brown) and counter-stained with hematoxylin (blue). Left: lower magnification (×40). Right: higher magnification (×100). Blue arrow: fibroblasts/mononuclear cells. Orange arrow: endothelial cells. **B** Double immunofluorescence staining with anti-MC1R antibody (green) and cell-specific markers (pink). Merged images with MC1R and each cell marker is shown. CD68: monocytes/macrophage; prolyl-4-hydroxylas β (P4Hβ): fibroblast; CD31; endothelial cells; CD66b: neutrophils. White circle: co-localized areas

Immunohistochemical analysis was performed to examine the presence of MC1R in skin biopsies of healthy donors and SSc patients. The characteristics of the donors are summarized in Table 1. Single staining showed that MC1R immunoreactivity was mainly observed in the fibroblasts/mononuclear cells, blood vessels, and epidermis of the skin from patients with dcSSc (Fig. 6A). For the purpose of qualitative evaluation, MC1R immunoreactivity was separately scored in four categories: total field, vessels, epidermis, and fibroblast/mononuclear cells (fibroblasts and monocytes were analyzed without distinction because they were indistinguishable by single staining). MC1R positivity was observed in 24 of 30 healthy donors, 40 of 50 dcSSc patients, and 9 of 10 lcSSc patients. MC1R score for staining intensity was similar across healthy, dcSSc, and lcSSc patients in all categories of tissue elements (Table 2; Fig. s2 shows individual plots). There was no apparent correlation between MC1R and modified Rodnan skin score (mRSS) in all categories of tissue elements (supplementary Fig. s3). These results indicate MC1R expression was frequently observed in SSc patients regardless of disease activity.

**Table 1:** Clinical characteristics of SSc patients in the cutaneous immunohistochemical analysis

Clinical information	Healthy	dcSSc	lcSSc
Total number	30	50	10
Sex (female:male)	21:9 (70%:30%)	35:15 (70%:30%)	8:2 (80%:20%)
Race	Caucasian (100%)	Caucasian (100%)	Caucasian (100%)
Age (years)^a^	46.7 ± 13.3	45.5 ± 11.9	61.2 ± 9.4
mRSS^a^	-	19.1 ± 4.4	8.1 ± 1.9
Disease duration (years)^a^	-	3.2 ± 2.2	7.7 ± 3.8
DMARD therapy	-	12 (24%)	1 (10%)
CRP elevation	-	18 (36%)	0 (0%)
Anti-Scl-70	-	15 (30%)	0 (0%)

Values are expressed as absolute number (%) unless otherwise indicated

*Abbreviations*: *dcSSc* diffuse cutaneous systemic sclerosis, *lcSSc* limited cutaneous systemic sclerosis, *mRSS* modified Rodnan skin score, *DMARD* disease-modifying anti-rheumatic drug, *CRP* C-reactive protein, *Anti-Scl-70* anti-topoisomerase I antibodies

^a^Values expressed as mean ± SEM

**Table 2:** Scores of melanocortin-1 receptor (MC1R) immunostaining of skin sections

IHC (MC1R staining) evaluation	Healthy	dcSSc	lcSSc
Total score	0.83 ± 0.13	0.94 ± 0.11	0.65 ± 0.17
Vessel score	0.70 ± 0.13	0.68 ± 0.08	0.75 ± 0.15
Fibroblast/mononuclear cell score	0.70 ± 0.12	0.89 ± 0.10	0.65 ± 0.17
Epidermis score	0.85 ± 0.17	0.83 ± 0.15	0.60 ± 0.19
Positive rate of MC1R expression^a^	24/30 (80%)	40/50 (80%)	9/10 (90%)

Scores are expressed as mean ± SEM (no staining, 0; faint staining, 0.5; light staining, 1; moderate staining, 2; dark staining, 3)

*Abbreviations*: *IHC* immunohistochemistry, *MC1R* melanocortin 1 receptor

^a^Values are expressed as an absolute number (%). MC1R positivity was defined as a total score of 0.5 or higher (faint staining)

To determine the cell type of MC1R-positive cells in patients with dcSSc, we used a double-staining protocol that co-stained MC1R with cell-specific markers. MC1R-positive cells were mainly monocytes/macrophages and there was staining of fibroblasts, endothelial cells, and neutrophils to a lesser extent (Fig. 6B).

## Discussion

**Fig. 7:**
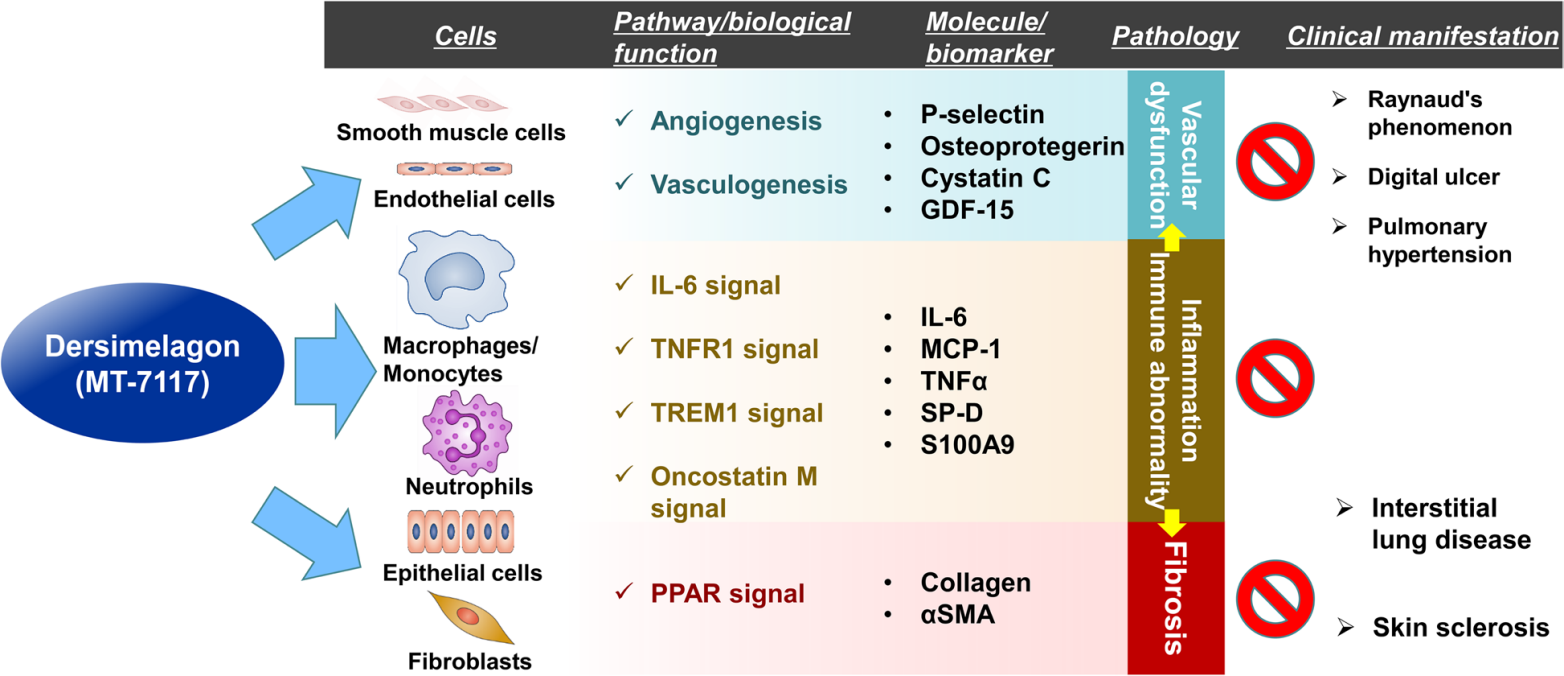
Mechanism of action of MT-7117 in systemic sclerosis. Schematic diagram illustrating the mechanism of MT-7117 based on findings of the present study

The results of the present study demonstrated that MT-7117 ameliorates skin fibrosis and lung inflammation in BLM-induced SSc murine models. The analyses of the mechanism of action of MT-7117 in the BLM-induced SSc model revealed that its primary action is anti-inflammatory via suppression of activation of inflammatory cells, such as monocytes/macrophages, and inflammation-related signals, such as IL-6 signaling. There was also evidence that MT-7117 suppressed vascular dysfunction by acting on endothelial cells. Moreover, MT-7117 showed an anti-fibrotic effect by suppressing fibroblast activation in vitro. Therefore, MT-7117 exerts disease-modifying effects with modulation of all three cardinal features of SSc, inflammation, vascular dysfunction, and fibrosis [6]. Based on these results, the mechanism of action of MT-7117 is schematically summarized in Fig. 7.

DNA microarray analysis performed to elucidate the mechanism of action of MT-7117 in the lung revealed that MT-7117 mainly targets inflammation-related categories and pathways. It is noteworthy that IL-6 itself and IL-6 pathway-related genes are strongly induced by BLM and markedly inhibited by MT-7117. IL-6 is an important inflammatory cytokine, and anti-IL-6 receptor or anti-IL-6 antibodies have been approved for the treatment of inflammatory diseases such as rheumatoid arthritis [39]. Recently, tocilizumab (Actemra®), an anti-IL-6 receptor antibody, was approved by the US FDA for SSc-associated ILD [9, 10]. Therefore, MT-7117, which strongly inhibits IL-6 signaling, is also expected to be effective against SSc-associated ILD. Additionally, it is interesting that the category of vascular dysfunction, which is the earliest and possibly the primary event in SSc [40], was extracted as the pathology targeted by MT-7117. Serum protein profiling showed that MT-7117 inhibited the expression of P-selectin and osteoprotegerin. These molecules are known to be associated with vascular dysfunction and are elevated in the plasma of patients with vascular damage in various diseases including SSc [41–44]. These results of serum protein profiling support those of DNA microarray that demonstrate the effect of MT-7117 on vascular dysfunction. As described in the “Background” section, it has been reported that αMSH, an in vivo ligand of MC1R, has anti-vascular dysfunction effects in several animal models [21–23]. These pieces of evidence suggest that MT-7117 is effective for conditions associated with SSc, such as Raynaud’s phenomenon, digital ulcer, and pulmonary hypertension, which are closely related to vascular dysfunction. The direct effects of MT-7117 on vascular endothelial and smooth muscle cells should be clarified in future studies. Moreover, serum protein profiling in the BLM model for therapeutic evaluation revealed that multiple factors were induced by BLM and suppressed by MT-7117. Notably, most of these factors are increased in the blood of SSc patients and correlate with SSc symptoms [36]: cystatin C correlates with right heart function in SSc patients [45]; GDF-15 correlates with organ involvement (particularly with lung disease) [46]; osteoprotegerin correlates with Medsger skin score [47] and increases in lcSSc patients [44]; P-selectin correlates with Health Assessment Questionnaire Disability Index [43]; S100A9 is high in patients with positive pulmonary fibrosis or autoantibodies [48]; TIMP-1 is high in serum [49]; and TNFRI and TNFRII are high in serum and T cells in skin [50]. Additionally, SP-D, which was suppressed by MT-7117 in prophylactic evaluation using the BLM-induced SSc model, is negatively correlated with pulmonary function [% diffusing capacity of the lungs for carbon monoxide and % vital capacity] in SSc patients [51]. These findings suggest similarities between the BLM model and SSc pathology, and the above factors are potential predictive and pharmacodynamic biomarkers for assessing MT-7117.

The suppression of human fibroblast activation by MT-7117 is considered to be an important result, demonstrating that MT-7117 exerts its anti-fibrotic effect by directly targeting fibroblasts and that it acts not only in animal models but also on human fibroblasts. Moreover, the effect of MT-7117 on skin fibroblasts derived from SSc patients and those from healthy donors was similar. This is an important result in the preclinical evaluation of MT-7117 for the treatment of SSc. MT-7117 has no inhibitory effect on collagen mRNA expression, which is consistent with previous reports evaluating αMSH in a similar assay system [52]. In this report, αMSH did not suppress the expression of *COL1A1* mRNA but suppressed the secretion of procollagen type I C-terminal peptide, suggesting the existence of a post-transcriptional mechanism such as inhibition of peptidase required for cleavage of C-terminal peptides from procollagen. Further studies are needed to elucidate the mechanism by which MT-7117 regulates collagen production.

To the best of our knowledge, our study is the first to investigate the distribution of MC1R expression at the protein level in skin samples from as many as 50 SSc patients. The analysis of MC1R expression by immunostaining confirmed that MC1R is expressed by monocytes/macrophages, neutrophils, blood vessels (endothelial cells), fibroblasts, and in the epidermis (keratinocytes) of the skin of SSc patients. Therefore, these MC1R-positive cells could be the targets of MT-7117, and this hypothesis would be consistent with the results of the investigation into the mechanism of action of MT-7117 using preclinical SSc models. Importantly, these cell types play a central role in inflammation, vascular dysfunction, and fibrosis, suggesting that the effect of MT-7117 on various target cells may synergistically contribute to its disease-modifying effects. However, the expression levels of MC1R varied among patients and were not detected in 20% and 10% of dcSSc and lcSSc patients, respectively. This raises the concern that the efficacy of MT-7117 may differ among individuals. The correlation between efficacy and expression level of MC1R should be verified in clinical trials using freshly prepared skin biopsy samples. The following points cannot be fully discussed due to the study limitations: (i) Since activation of MC1R signaling is known to suppress fibrosis in SSc pathology [24–26], it is predicted that the ligand expression is decreased and/or the receptor signal is attenuated in SSc patients compared with healthy subjects. Unfortunately, the expression level of αMSH was not evaluated in the present study. (ii) Since the evaluation of cell types expressing MC1R by double staining was performed in a small number of samples, the present study was limited to a qualitative evaluation of MC1R expression in each cell type. Further evaluation will be necessary to address the above issues.

## Conclusions

In summary, the findings in this study demonstrate that MT-7117 exerts its positive effects by affecting the pathologies of inflammation, vascular dysfunction, and fibrosis through its pleiotropic function on inflammatory cells, endothelial cells, and fibroblasts. In view of its potent beneficial impact on all three cardinal pathologies of SSc, MT-7117 is a potential therapeutic agent for the treatment of clinically challenging SSc, which has diverse and difficult to treat symptoms. A phase 2 clinical trial investigating the efficacy and tolerability of MT-7117 in patients with diffuse cutaneous SSc is currently in progress (NCT04440592).

## Supplementary Information

**Additional file 1: Fig. s1.** Serum protein profiling of the BLM-induced SSc model treated with MT-7117. Serum samples from BLM-induced SSc mouse model (therapeutic model) treated with MT-7117 at 10 mg/kg or imatinib at 150 mg/kg from were used for serum protein profiling (all proteins that could be quantified). Using Luminex® assays, 110 proteins were investigated. The graphs show 67 proteins that were detectable in the quantitative range. All values are expressed as an individual plot dot and the mean ± SEM (*n* = 8). * *p* < 0.05, ** *p* < 0.01 by Student’s t-test (vs. BLM_vehicle group).

**Additional file 2: Fig. s2.** Scores of melanocortin-1 receptor (MC1R) immunostaining in skin sections. The staining intensity of MC1R in skin samples from healthy subjects (*n* = 30), dcSSc patients (*n* = 50), and lcSSc (*n* = 10) patients was graded from 0 to 3. Qualitative assessment was performed by assigning a score based on staining intensity after identifying each stained cell type and tissue element. No staining, 0; faint staining, 0.5; light staining, 1; moderate staining, 2; dark staining: 3. All values are expressed as an individual plot dot and the mean ± SEM.

**Additional file 3: Fig. s3.** Correlation between MC1R scores and mRSS in dcSSc patients. Staining intensity of MC1R in the skin of dcSSc patients (*n* = 50) was graded from 0 to 3. MC1R scores and mRSS of dcSSc patients were plotted, and the coefficient of determination (R^2^) was calculated. All values are expressed as an individual plot dot.

**Additional file 4: Table s1.** List of proteins measured in serum protein profiling.

**Additional file 5: Table s2.** Microarray-based gene expression profiling of the BLM-induced SSc model treated with MT-7117.

## Data Availability

The datasets generated or analyzed during in the current study are available from the corresponding author upon reasonable request. The microarray data used in the current study is available in the National Center for Biotechnology Information Gene Expression Omnibus database (GSE 199581).

## Abbreviations

MC1R: Melanocortin 1 receptor
SSc: Systemic sclerosis
BLM: Bleomycin
TGF-β: Transforming growth factor-β
p.o.: Oral administration
αSMA: α-Smooth muscle actin (encoded by *ACTA2*)
FDA: Food and Drug Administration
ILD: Interstitial lung disease
αMSH: α-Melanocyte-stimulating hormone
TNF-α: Tumor necrosis factor-α
IL: Interleukin
KC: Keratinocyte-derived chemokine
dcSSc: Diffuse cutaneous SSc
DMSO: Dimethylsulfoxide
PBS: Phosphate-buffered saline
SP-D: Surfactant protein D
Ccl2: Chemokine CC ligand-2
MCP-1: Monocyte chemotactic protein-1
Hprt: Hypoxanthine phosphoribosyltransferase
CT: Cycle threshold
HE: Hematoxylin and eosin
DEGs: Differentially expressed genes
DSEA: Downstream effect analysis
DMEM: Dulbecco’s modified Eagle medium
FCS: Fetal calf serum
EDTA: Ethylene diamine tetra-acetic acid
GAPDH: Glyceraldehyde-3-phosphate dehydrogenase
COL1A1: Collagen type I alpha 1
lcSSc: Limited cutaneous SSc
SEM: Standard error of the mean
EC_50_: Half maximal effective concentration
TREM-1: Triggering receptor expressed on myeloid cells-1
PPAR: Peroxisome proliferator-activated receptor
GDF-15: Growth and differentiation factor-15
MMP: Matrix metalloproteinase
TNFR: Tumor necrosis factor receptor
TIMP-1: Tissue inhibitor of metalloproteinases-1
mRSS: Modified Rodnan skin score
