# Supplementary material for: Dersimelagon, a novel oral melanocortin 1 receptor agonist, demonstrates disease-modifying effects in preclinical models of systemic sclerosis
Source: Arthritis Res Ther. 2022 Sep 1;24:210. doi: 10.1186/s13075-022-02899-3 (PMC9434962; doi:10.1186/s13075-022-02899-3)
Supplement: Supplementary file 1 — Additional file 1: Fig. s1. Serum protein profiling of the BLM-induced SSc model treated with MT-7117. Serum samples from BLM-induced SSc mouse model (therapeutic model) treated with MT-7117 at 10 mg/kg or imatinib at 150 mg/kg from were used for serum protein profiling (all proteins that could be quantified). Using Luminex® assays, 110 proteins were investigated. The graphs show 67 proteins that were detectable in the quantitative range. All values are expressed as an individual plot dot and the mean ± SEM (n = 8). * p < 0.05, ** p < 0.01 by Student’s t-test (vs. BLM_vehicle group). [file 13075_2022_2899_MOESM1_ESM.pptx]

## Slide 1
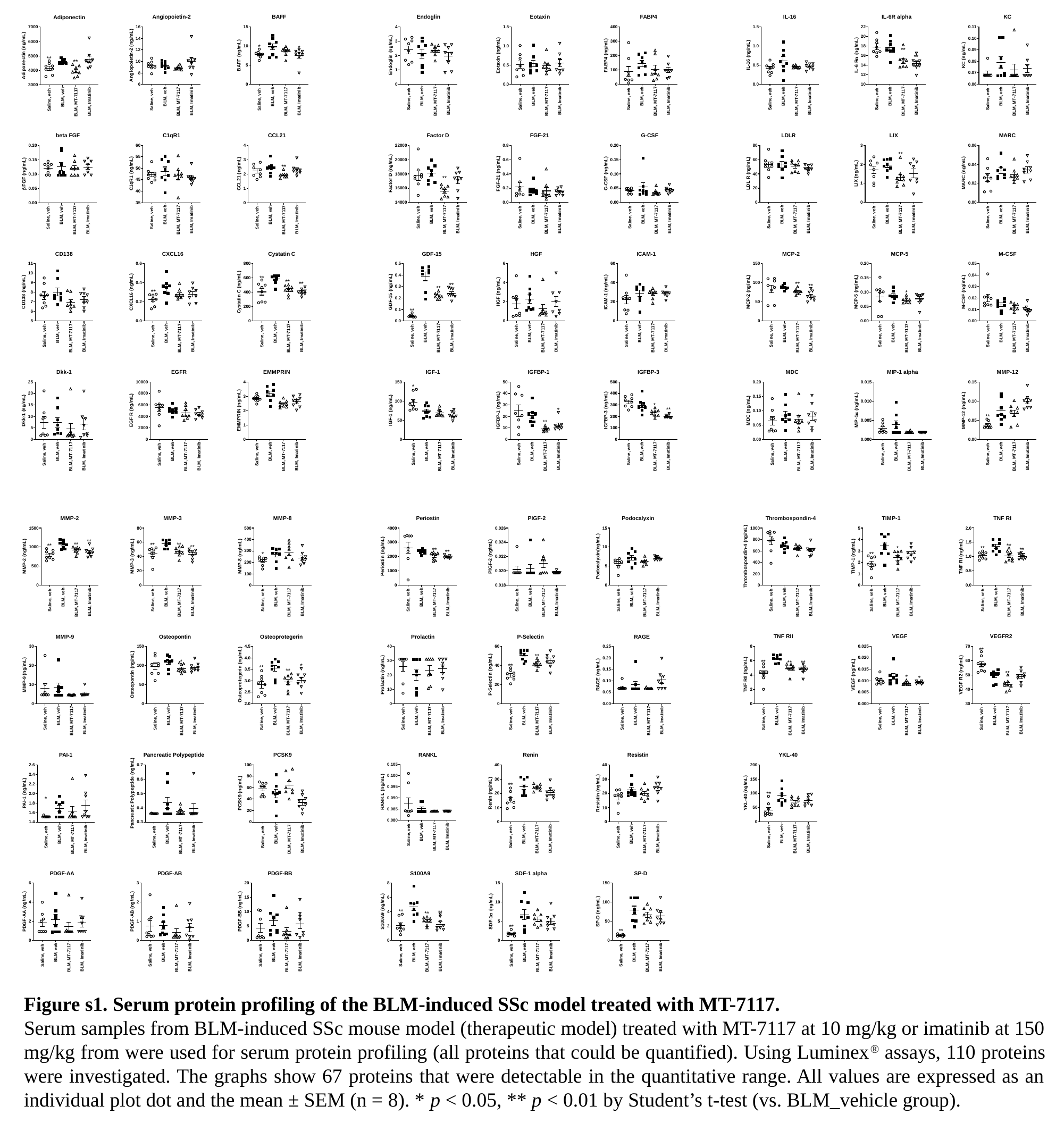

Figure s1. Serum protein profiling of the BLM-induced SSc model treated with MT-7117.
Serum samples from BLM-induced SSc mouse model (therapeutic model) treated with MT-7117 at 10 mg/kg or imatinib at 150 mg/kg from were used for serum protein profiling (all proteins that could be quantified). Using Luminex® assays, 110 proteins were investigated. The graphs show 67 proteins that were detectable in the quantitative range. All values are expressed as an individual plot dot and the mean ± SEM (n = 8). * p < 0.05, ** p < 0.01 by Student’s t-test (vs. BLM_vehicle group).
