# Supplementary material for: Dersimelagon, a novel oral melanocortin 1 receptor agonist, demonstrates disease-modifying effects in preclinical models of systemic sclerosis
Source: Arthritis Res Ther. 2022 Sep 1;24:210. doi: 10.1186/s13075-022-02899-3 (PMC9434962; doi:10.1186/s13075-022-02899-3)
Supplement: Supplementary file 2 — Additional file 2: Fig. s2. Scores of melanocortin-1 receptor (MC1R) immunostaining in skin sections. The staining intensity of MC1R in skin samples from healthy subjects (n = 30), dcSSc patients (n = 50), and lcSSc (n = 10) patients was graded from 0 to 3. Qualitative assessment was performed by assigning a score based on staining intensity after identifying each stained cell type and tissue element. No staining, 0; faint staining, 0.5; light staining, 1; moderate staining, 2; dark staining: 3. All values are expressed as an individual plot dot and the mean ± SEM. [file 13075_2022_2899_MOESM2_ESM.pptx]

## Slide 1
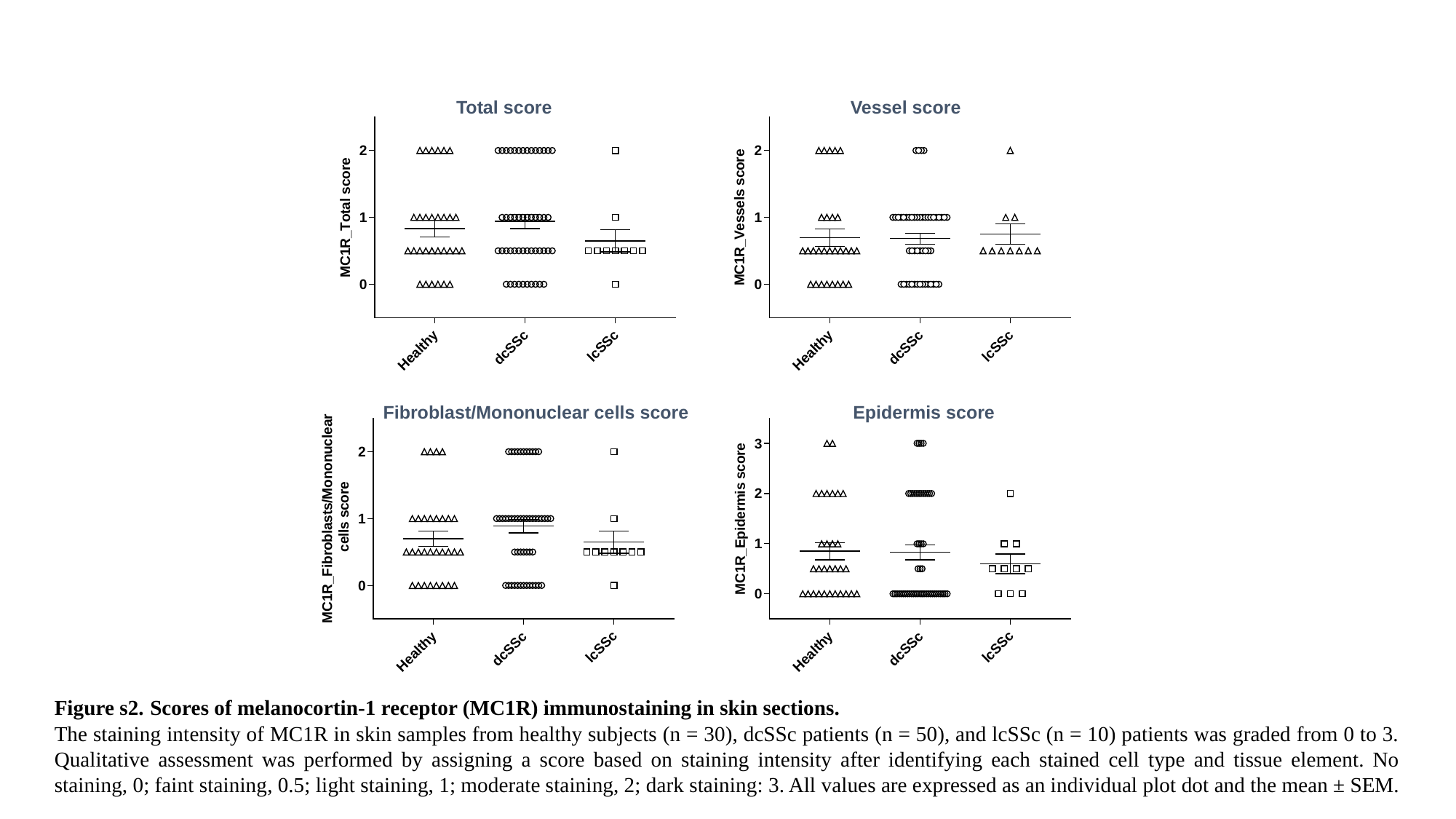

Total score
Vessel score
Fibroblast/Mononuclear cells score
Epidermis score
Figure s2. Scores of melanocortin-1 receptor (MC1R) immunostaining in skin sections.
The staining intensity of MC1R in skin samples from healthy subjects (n = 30), dcSSc patients (n = 50), and lcSSc (n = 10) patients was graded from 0 to 3. Qualitative assessment was performed by assigning a score based on staining intensity after identifying each stained cell type and tissue element. No staining, 0; faint staining, 0.5; light staining, 1; moderate staining, 2; dark staining: 3. All values are expressed as an individual plot dot and the mean ± SEM.
