# Supplementary material for: Dersimelagon, a novel oral melanocortin 1 receptor agonist, demonstrates disease-modifying effects in preclinical models of systemic sclerosis
Source: Arthritis Res Ther. 2022 Sep 1;24:210. doi: 10.1186/s13075-022-02899-3 (PMC9434962; doi:10.1186/s13075-022-02899-3)
Supplement: Supplementary file 3 — Additional file 3: Fig. s3. Correlation between MC1R scores and mRSS in dcSSc patients. Staining intensity of MC1R in the skin of dcSSc patients (n = 50) was graded from 0 to 3. MC1R scores and mRSS of dcSSc patients were plotted, and the coefficient of determination (R2) was calculated. All values are expressed as an individual plot dot. [file 13075_2022_2899_MOESM3_ESM.pptx]

## Slide 1
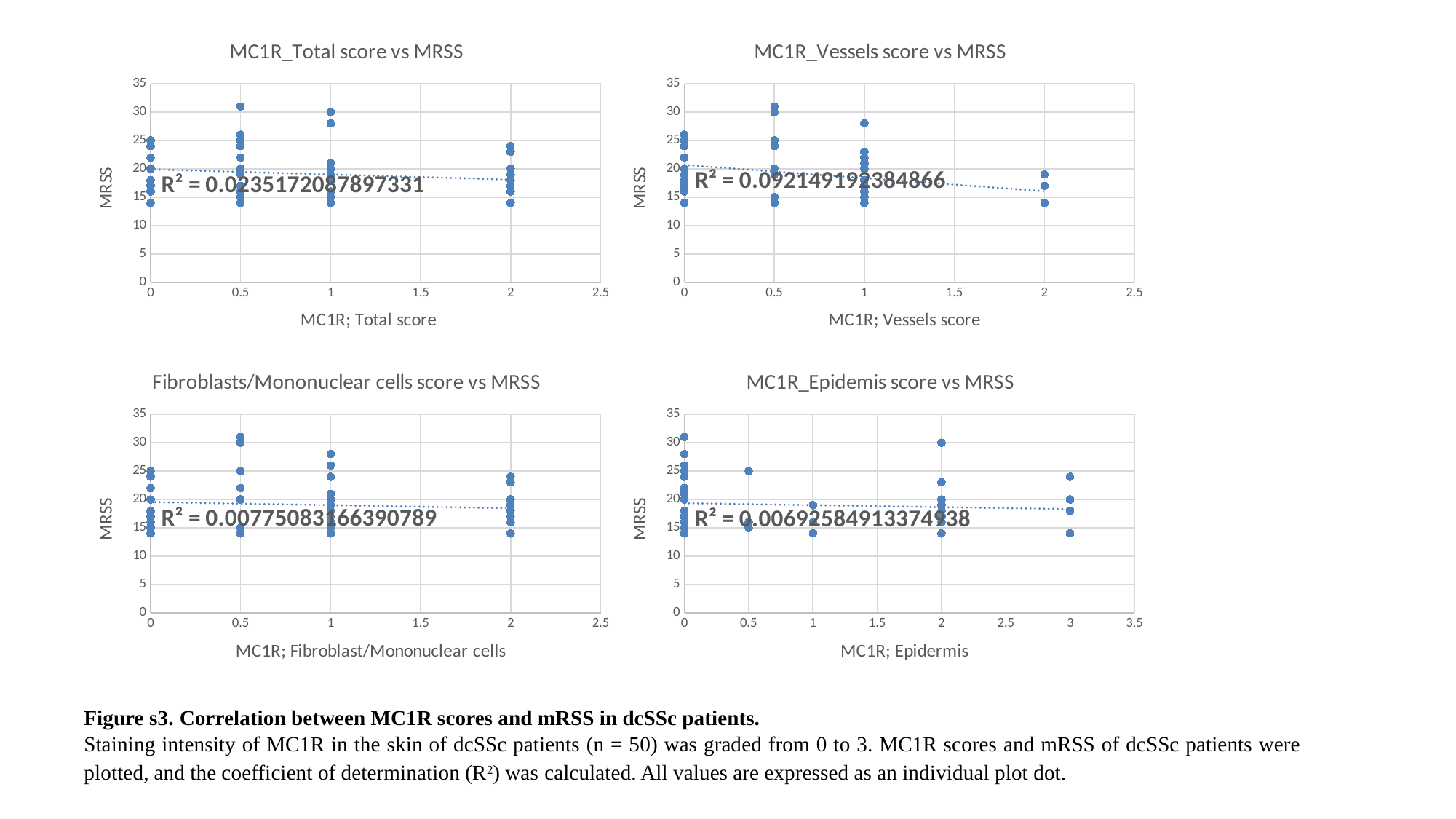

### Chart: MC1R_Total score vs MRSS
| Category | |
|---|---|
### Chart: MC1R_Vessels score vs MRSS
| Category | |
|---|---|
### Chart: Fibroblasts/Mononuclear cells score vs MRSS
| Category | |
|---|---|
### Chart: MC1R_Epidemis score vs MRSS
| Category | |
|---|---|Figure s3. Correlation between MC1R scores and mRSS in dcSSc patients.
Staining intensity of MC1R in the skin of dcSSc patients (n = 50) was graded from 0 to 3. MC1R scores and mRSS of dcSSc patients were plotted, and the coefficient of determination (R2) was calculated. All values are expressed as an individual plot dot.
