# Supplementary material for: Dersimelagon, a novel oral melanocortin 1 receptor agonist, demonstrates disease-modifying effects in preclinical models of systemic sclerosis
Source: Arthritis Res Ther. 2022 Sep 1;24:210. doi: 10.1186/s13075-022-02899-3 (PMC9434962; doi:10.1186/s13075-022-02899-3)
Supplement: Supplementary file 4 — Additional file 4: Table s1. List of proteins measured in serum protein profiling. [file 13075_2022_2899_MOESM4_ESM.docx]

**Table s1.** **List of proteins measured in serum protein profiling**

| Protein name |
| --- |
|  |
| Adiponectin |
| Haptoglobin |
| IGFBP-3 (insulin-like growth factor-binding protein 3) |
| FABP4 (fatty acid binding protein 4) |
| YKL-40 (also known as chitinase-3-like-1) |
| PDGF-BB (platelet-derived growth factor) |
| Resistin |
| PAI-1 (plasminogen activator inhibitor-1) |
| IGF-1 (insulin-like growth factor-1) |
| Osteopontin |
| MMP-9 (matrix metalloproteinase-9) |
| CCL21 (chemokine (C-C motif) ligand 21) |
| Thrombospondin-4 |
| PDGF-AA |
| EGFR (epidermal growth factor receptor) |
| Cystatin C |
| IGFBP-1 |
| MMP-2 |
| MCP-2 (monocyte chemoattractant protein-2) |
| MMP-3 |
| CRP (C-reactive protein) |
| Periostin |
| Factor D |
| HGF (hepatocyte growth factor) |
| IL-12 p70 (interleukin-12 p70) |
| IL-6 R alpha (interleukin-6 receptor alpha) |
| EMMPRIN (extracellular matrix metalloproteinase inducer) |
| S100A8 |
| S100A9 |
| RANKL (receptor activator of NF-κB ligand) |
| VEGFR2 (Vascular endothelial growth factor receptor 2) |
| Leptin |
| G-CSF (granulocyte-colony stimulating factor) |
| TIMP-1 (tissue inhibitor of metalloproteinases-1) |
| PDGF-AB |
| TIMP-4 |
| RAGE (receptor for advanced glycation end products) |
| GM-CSF (granulocyte macrophage colony-stimulating factor) |
| TNF-alpha (tumor necrosis factor-alpha) |
| TNF RI (tumor necrosis factor receptor 1) |
| IL-1 beta |
| VEGF |
| IL-2 |
| IL-4 |
| IL-5 |
| IL-6 |
| IFN-gamma (interferon-gamma) |
| IL-3 |
| IL-16 |
| LDLR (low density lipoprotein receptor) |
| Nephrin |
| Osteoprotegerin |
| IL-23 p19 |
| IL-33 |
| Prolactin |
| Pancreatic Polypeptide |
| P-Selectin |
| Renin |
| CD138 |
| TWEAK (Tumor necrosis factor-like weak inducer of apoptosis) |
| MMP-8 |
| Oncostatin M |
| TNF RII |
| Podocalyxin |
| SP-D (surfactant protein D) |
| KC (keratinocyte chemoattractant) |
| IL-7 |
| GDF-15 (growth and differentiation factor-15) |
| MCP-1 |
| MIP-3 beta (macrophage inflammatory protein-3) |
| MIP-2 |
| CXCL13 (C-X-C motif chemokine ligand 13) |
| CXCL16 |
| DPPIV (dipeptidyl peptidase-4) |
| EGF |
| Fas Ligand |
| IL-10 |
| IL-13 |
| IL-17A |
| Endoglin |
| Glucagon |
| ICAM-1 (intercellular adhesion molecule-1) |
| IP-10 (interferon gamma-induced protein-10) |
| RANTES (regulated on activation, normal T cell expressed and secreted) |
| MARC (also known as CCL7) |
| MCP-5 |
| beta-NGF (beta nerve growth factor) |
| PlGF-2 (placental growth factor) |
| M-CSF (macrophage colony stimulating factor) |
| MIP-1 alpha |
| IL-1 alpha |
| MIP-3 alpha |
| MIP-1 beta |
| PCSK9 (proprotein convertase subtilisin/kexin type 9) |
| SDF-1 alpha (stromal cell-derived factor 1) |
| IL-17E |
| IL-27 |
| BAFF (B cell activating factor belonging to the tumor necrosis factor family) |
| FGF-21 (fibroblast growth factor-21) |
| Granzyme B |
| C1qR1 |
| beta-FGF |
| LIX (lipopolysaccharide-induced CXC chemokine) |
| Angiopoietin-2 |
| KIM-1 (kidney injury molecule-1) |
| MMP-12 |
| Eotaxin |
| MDC (macrophage-derived chemokine) |
| uPAR (urokinase-type plasminogen activator receptor) |
| Dkk-1 (dickkopf1) |
