# Supplementary material for: Dersimelagon, a novel oral melanocortin 1 receptor agonist, demonstrates disease-modifying effects in preclinical models of systemic sclerosis
Source: Arthritis Res Ther. 2022 Sep 1;24:210. doi: 10.1186/s13075-022-02899-3 (PMC9434962; doi:10.1186/s13075-022-02899-3)
Supplement: Supplementary file 5 — Additional file 5: Table s2. Microarray-based gene expression profiling of the BLM-induced SSc model treated with MT-7117. [file 13075_2022_2899_MOESM5_ESM.docx]

**Table s2.** **Microarray-based gene expression profiling of the BLM-induced SSc model treated with MT-7117**

(A) Categories by cell types that changed in the BLM-induced SSc model treated with MT-7117

| Cell type | Diseases or functions | Activation z-score [BLM] vs [PBS] | Activation z-score [BLM_7117] vs [BLM] | *p*-value [BLM] vs [PBS] | *p*-value [BLM_7117] vs [BLM] |
| --- | --- | --- | --- | --- | --- |
| Macrophages | Activation | 3.727 | -3.912 | 1.30E-14 | 1.20E-15 |
|  | Cell movement | 3.329 | -2.615 | 8.56E-18 | 4.31E-21 |
|  | Chemotaxis | NS | -2.728 | NS | 6.45E-12 |
|  | Immune response | NS | -2.697 | NS | 7.90E-12 |
|  | Migration | NS | -2.519 | NS | 2.29E-13 |
|  | Phagocytosis | NS | -3.231 | NS | 8.75E-11 |
|  | Recruitment | NS | -3.505 | NS | 2.64E-14 |
|  | Response | NS | -2.702 | NS | 1.64E-11 |
| Mononuclear leukocytes | Activation | 3.500 | -3.478 | 4.00E-16 | 1.76E-21 |
|  | Cell movement | 3.089 | -3.441 | 2.28E-22 | 7.57E-26 |
|  | Chemotaxis | 3.856 | -3.709 | 1.93E-15 | 2.80E-20 |
|  | Hematopoiesis | 2.014 | -3.270 | 6.07E-14 | 1.42E-14 |
|  | Homing | 3.841 | -3.727 | 2.77E-14 | 5.44E-19 |
|  | Migration | 3.288 | -3.847 | 4.06E-19 | 2.83E-22 |
|  | Recruitment | 2.240 | -2.900 | 6.92E-14 | 1.03E-21 |
|  | Response | 2.591 | -2.423 | 7.66E-15 | 5.59E-24 |
|  | Homeostasis | NS | -3.332 | NS | 5.65E-12 |
|  | Binding | NS | -3.409 | NS | 3.19E-10 |
|  | Interaction | NS | -3.675 | NS | 3.73E-11 |
| Neutrophils | Cell movement | 3.147 | -2.669 | 9.19E-26 | 7.07E-27 |
|  | Chemotaxis | 2.385 | -2.908 | 1.58E-16 | 5.10E-16 |
|  | Degranulation | 2.630 | -2.219 | 3.56E-14 | 7.92E-11 |
|  | Recruitment | 2.932 | -3.195 | 7.95E-20 | 3.69E-23 |
|  | Accumulation | NS | -2.489 | NS | 9.63E-11 |
|  | Activation | NS | -2.318 | NS | 2.57E-11 |
|  | Immune response | NS | -1.937 | NS | 5.51E-13 |
|  | Migration | NS | -2.494 | NS | 1.86E-13 |
|  | Phagocytosis | NS | -2.018 | NS | 1.13E-09 |
| Smooth muscle cells | Cell movement | 2.908 | -2.696 | 6.31E-13 | 2.48E-18 |
|  | Proliferation | 3.494 | -2.597 | 9.20E-18 | 8.94E-18 |
|  | Migration | NS | -2.235 | NS | 1.06E-16 |
| Epithelial cells | Proliferation | 2.166 | -2.590 | 1.14E-19 | 1.59E-14 |
| T lymphocytes | Activation | NS | -3.326 | NS | 3.29E-14 |
|  | Cell movement | 1.957 | -3.195 | 1.51E-15 | 5.57E-19 |
|  | Chemotaxis | NS | -3.539 | NS | 4.89E-13 |
|  | Homing | NS | -3.405 | NS | 8.47E-12 |
|  | Recruitment | NS | -2.170 | NS | 7.23E-15 |
| Endothelial cells | Binding | NS | -3.302 | NS | 2.08E-10 |

NS = not significant.

Analyses were performed using differentially expressed genes from disease models ([BLM] vs [PBS]) and MT-7117 treatment ([MT_7117] vs [BLM]). Ingenuity pathway analysis (IPA) was used to calculate the activation z-score. The activation z-score is a predicted score of functional activation/inhibition defined by IPA. The positive z-score indicates an expected change in activation and a negative z-score indicates an expected change in the inhibition.

(B) Categories related to inflammation, immune abnormality, and vasculopathy that changed in the BLM-induced SSc model treated with MT-7117

|  | Diseases or functions | Activation z-score [BLM] vs [PBS] | Activation z-score [BLM_7117] vs [BLM] | *p*-value [BLM] vs [PBS] | *p*-value [BLM_7117] vs [BLM] |
| --- | --- | --- | --- | --- | --- |
| Inflammation | Inflammatory response | 3.566 | -4.080 | 5.84E-48 | 3.17E-50 |
| Immune abnormality | Activation of antigen presenting cells | 3.383 | -3.943 | 1.88E-16 | 2.39E-20 |
| Vasculopathy | Angiogenesis | 2.694 | -2.873 | 2.36E-22 | 8.43E-19 |
|  | Atherosclerosis | 2.843 | -3.065 | 3.87E-26 | 5.47E-21 |
|  | Vasculogenesis | 2.509 | -2.924 | 1.54E-20 | 9.02E-19 |
|  | Vaso-occlusion | 2.911 | -3.115 | 1.06E-27 | 1.57E-22 |

Analyses were performed using differentially expressed genes from disease models ([BLM] vs [PBS]) and MT-7117 treatment ([MT_7117] vs [BLM]). Ingenuity pathway analysis (IPA) was used to calculate the activation z-score. The activation z-score is a predicted score of functional activation/inhibition defined by IPA. The positive z-score indicates an expected change in activation and a negative z-score indicates an expected change in the inhibition.

(C) Signaling pathways related to inflammation and fibrosis that changed in the BLM-induced SSc model treated with MT-7117

|  | Canonical pathways | Activation z-score [BLM] vs [PBS] | Activation z-score [BLM_7117] vs [BLM] | *p*-value [BLM] vs [PBS] | *p*-value [BLM_7117] vs [BLM] |
| --- | --- | --- | --- | --- | --- |
| Inflammation | TREM1 Signaling | 2.828 | -2.496 | 5.01E-05 | 3.72E-06 |
|  | IL-6 Signaling | 2.600 | -2.673 | 1.91E-05 | 6.76E-05 |
|  | Oncostatin M Signaling | 2.121 | -2.236 | 2.82E-02 | 2.14E-02 |
|  | Aryl Hydrocarbon Receptor Signaling | 2.683 | -1.414 | 3.80E-06 | 2.75E-03 |
|  | p38 MAPK Signaling | 2.000 | -1.000 | 2.40E-02 | 3.31E-02 |
| Fibrosis | PPAR Signaling | -2.828 | 2.121 | 3.24E-03 | 4.07E-02 |

Analyses were performed using differentially expressed genes from disease models ([BLM] vs [PBS]) and MT-7117 treatment ([MT_7117] vs [BLM]). Ingenuity pathway analysis (IPA) was used to calculate the activation z-score. The activation z-score is a predicted score of functional activation/inhibition defined by IPA. The positive z-score indicates an expected change in activation and a negative z-score indicates an expected change in the inhibition.

(D) Fluctuating genes involved in IL-6 signaling

| Symbol | Entrez Gene name | Fold change [BLM] vs [PBS] | *p*-value [BLM] vs [PBS] | Fold change [BLM_7117] vs [BLM] | p-value [BLM_7117] vs [BLM] |
| --- | --- | --- | --- | --- | --- |
| ABCB1 | ATP binding cassette subfamily B member 1 | 2.869 | 1.49E-03 | -1.734 | 1.94E-03 |
| CD14 | CD14 molecule | 2.432 | 8.06E-13 | -1.702 | 2.98E-07 |
| COL1A1 | collagen type I alpha 1 chain | 2.937 | 7.10E-16 | -1.534 | 7.05E-03 |
| FOS | Fos proto-oncogene, AP-1 transcription factor subunit | 2.154 | 9.99E-09 | -1.706 | 5.14E-06 |
| HRAS | HRas proto-oncogene, GTPase | -2.061 | 3.19E-08 | 1.366 | 3.00E-03 |
| IKBKE | inhibitor of nuclear factor kappa B kinase subunit epsilon | 2.836 | 1.12E-09 | -1.452 | 4.47E-03 |
| IL18 | interleukin 18 | -2.861 | 2.12E-15 | 1.722 | 1.68E-06 |
| IL18RAP | interleukin 18 receptor accessory protein | 2.121 | 4.98E-11 | -1.388 | 1.01E-03 |
| IL1B | interleukin 1 beta | 2.478 | 3.41E-10 | -2.520 | 1.63E-09 |
| IL1R2 | interleukin 1 receptor type 2 | 5.954 | 2.68E-16 | -2.443 | 2.13E-06 |
| IL1RL2 | interleukin 1 receptor like 2 | 2.171 | 3.43E-14 | -1.213 | 2.23E-02 |
| IL1RN | interleukin 1 receptor antagonist | 5.286 | 2.50E-17 | -1.495 | 7.62E-03 |
| IL36G | interleukin 36 gamma | 2.258 | 3.01E-06 | -1.530 | 4.06E-02 |
| IL6 | interleukin 6 | 26.185 | 1.24E-13 | -6.483 | 1.26E-07 |
| NFKBID | NFKB inhibitor delta | -2.075 | 1.70E-07 | 1.153 | 1.56E-01 |
| NFKBIE | NFKB inhibitor epsilon | 2.280 | 8.35E-15 | -1.658 | 1.57E-06 |
| NGFR | nerve growth factor receptor | 2.412 | 1.16E-11 | -1.449 | 4.51E-04 |
| PIK3C2G | phosphatidylinositol-4-phosphate 3-kinase catalytic subunit type 2 gamma | -3.825 | 2.14E-08 | 1.241 | 2.59E-01 |
| PIK3CD | phosphatidylinositol-4,5-bisphosphate 3-kinase catalytic subunit delta | 2.080 | 2.02E-10 | -1.835 | 2.80E-08 |
| PIK3R5 | phosphoinositide-3-kinase regulatory subunit 5 | 2.311 | 2.49E-05 | -1.360 | 3.36E-02 |
| RAP2B | RAP2B, member of RAS oncogene family | 2.573 | 1.51E-12 | -1.370 | 1.26E-03 |
| SOCS3 | suppressor of cytokine signaling 3 | 2.918 | 1.35E-14 | -2.037 | 7.50E-07 |
| TNF | tumor necrosis factor | 3.408 | 6.12E-09 | -1.870 | 1.02E-03 |
| TNFAIP6 | TNF alpha induced protein 6 | 3.424 | 3.47E-10 | -2.221 | 1.05E-07 |
| TNFRSF1B | TNF receptor superfamily member 1B | 2.749 | 6.48E-15 | -1.580 | 8.79E-04 |
| VEGFA | vascular endothelial growth factor A | -2.015 | 4.29E-15 | 1.234 | 2.31E-03 |

Analyses were performed using differentially expressed genes from disease models ([BLM] vs [PBS]) and MT-7117 treatment ([MT_7117] vs [BLM]).
